# Transcriptional responses of *Medicago truncatula* upon sulfur deficiency stress and arbuscular mycorrhizal symbiosis

**DOI:** 10.3389/fpls.2014.00680

**Published:** 2014-12-02

**Authors:** Daniel Wipf, Gaëlle Mongelard, Diederik van Tuinen, Laurent Gutierrez, Leonardo Casieri

**Affiliations:** ^1^UMR 1347 Agroécologie, Pôle Interactions Plantes-Microorganismes - ERL 6300 CNRS, Université de BourgogneDijon, France; ^2^CRRBM and BIOPI EA3900, Université de Picardie Jules VerneAmiens, France; ^3^Institut National de la Recherche Agronomique, UMR 1347 Agroécologie, Pôle Interactions Plantes-Microorganismes - ERL 6300 CNRSDijon, France

**Keywords:** *Medicago truncatula*, transcriptome, S deficiency, AM interaction, *Rhizophagus irregularis*

## Abstract

Sulfur plays an essential role in plants' growth and development and in their response to various abiotic and biotic stresses despite its leachability and its very low abundance in the only form that plant roots can uptake (sulfate). It is part of amino acids, glutathione (GSH), thiols of proteins and peptides, membrane sulfolipids, cell walls and secondary products, so reduced availability can drastically alter plant growth and development. The nutritional benefits of symbiotic interactions can help the plant in case of S deficiency. In particular the arbuscular mycorrhizal (AM) interaction improves N, P, and S plant nutrition, but the mechanisms behind these exchanges are not fully known yet. Although the transcriptional changes in the leguminous model plant *Medicago truncatula* have been already assessed in several biotic and/or abiotic conditions, S deficiency has not been considered so far. The aim of this work is to get a first overview on S-deficiency responses in the leaf and root tissues of plants interacting with the AM fungus *Rhizophagus irregularis*. Several hundred genes displayed significantly different transcript accumulation levels. Annotation and GO ID association were used to identify biological processes and molecular functions affected by sulfur starvation. Beside the beneficial effects of AM interaction, plants were greatly affected by the nutritional status, showing various differences in their transcriptomic footprints. Several pathways in which S plays an important role appeared to be differentially affected according to mycorrhizal status, with a generally reduced responsiveness to S deficiency in mycorrhized plants.

## Introduction

Sulfur is an essential macronutrient for photosynthetic organisms' growth and development, and for their response to various abiotic and biotic stresses. Plants use sulfate as a major S source to synthesize various essential molecules and thus sustain cell growth and viability (Saito, 2004). S is part of amino acids, glutathione (GSH), thiols of proteins and peptides, membrane sulfolipids, cell walls and secondary products like vitamins, cofactors and hormones (Foyer and Noctor, 2009; Popper et al., 2011), so reduced availability can have dramatic impacts on plant growth and development.

During the symbiotic AM interaction, the fungal symbiont plays an important role in plant nutrition mainly by supplying the plant with different nutrients and water in exchange for photosynthesized sugars (Ferrol and Pérez-Tienda, 2009; Smith and Smith, 2012). The crosstalk with the mycobiont triggers a series of events in the plant. For instance, specific mycorrhizal uptake mechanisms are activated, or constitutively-expressed transporters of the direct uptake mechanism are induced to increase the exchange and reallocation of nutrients such as P, N and S (Harrison et al., 2002; Hildebrandt et al., 2002; Paszkowski et al., 2002; Govindarajulu et al., 2005; Güimil et al., 2005; Karandashov and Bucher, 2005; Maeda et al., 2006; Javot et al., 2007; Sawers et al., 2008; Guether et al., 2009; Kobae et al., 2010; Smith and Smith, 2011; Smith et al., 2011; Casieri et al., 2013). The AM interaction involves nutrient-related benefits for the two symbionts and metabolic responses associated to nutrient exchanges. Moreover, the key steps of the establishment and maintenance of a functional symbiotic interaction involve massive cytological and metabolic rearrangements that are finely tuned by the regulation of the expression of a great number of genes (Smith and Read, 2008).

Genetic approaches have evidenced a fair number of plant genes required for AM or nodule symbiosis (Parniske, 2008) and for nutritional and developmental responses. Transcriptome analysis using cDNA arrays is a powerful approach to identify plant genes that are regulated upon a physiological, nutritional, pathogenic, or symbiotic condition.

Quite a few studies have already used transcriptome analyses to identify *Medicago truncatula* genes differentially expressed (DEGs) in various tissues or developmental stages (Benedito et al., 2008, 2010) or involved in AM/nodule symbiosis (Liu et al., 2003; Manthey et al., 2004; Hohnjec et al., 2005; Limpens et al., 2013). A comprehensive *M. truncatula* gene expression atlas (MtGEA), based on Affymetrix GeneChips designed on the genome annotation Mt1.0, is available on the Samuel Roberts Noble Foundation website (http://mtgea.noble.org/v3/index.php). Although an extensive transcript dataset is available from anatomical, growth-related, biological, physical, nutritional, and chemical treatments, a transcriptome analysis during S deficiency in non-mycorrhized and mycorrhized plants is still missing.

In a previous work we elucidated the role of *Medicago truncatula* sulfate transporter when plants respond to S starvation and how the AM interaction might help prevent stresses due to reduced sulfate availability (Casieri et al., 2012). In the present work we aim to get a first overview on the transcriptional responses of this model leguminous plant during the interaction with the AM fungus *Rhizophagus irregularis*, in normal (+S) or deficient (−S) sulfur availability conditions, by using a custom-designed Nimblegen microarray based on the latest annotation version (Mt3.5).

## Materials and methods

### Plant growth conditions and inoculation with the AM fungus

The factorial design of our experiments (2 × 2) included two S levels and two symbiotic conditions using *Medicago truncatula* cv *Jemalong*, line A17. The plants were grown in phytochambers with a 16/8 h day/night cycle (photon flux density between 350 and 400 μEm^−2^s^−1^) and temperatures of 23/21°C, respectively. Medicago seeds were chemically scarified by a 5 min sulfuric acid treatment, washed thoroughly under tap water, surface-Sterilized for 10 min in a 3.5% sodium hypochlorite solution and washed repeatedly with sterile de-ionized water until no chlorine smell was detectable. The sterilized seeds were placed on Whatman paper in 15 cm Petri dishes and then hydrated with de-ionized water at 4°C in the dark for 2–3 days. Then the Petri dishes were left at 25 ± 1°C under an 18/6 h day/night cycle for the seeds to germinate. After 3–4 days, plantlets with their sprouting first leaf were transplanted into 200 ml pots containing washed and sterilized quartz sand (0.8–2 mm) and inoculated with the AM fungus. The mycorrhizal fungus *Rhizophagus irregularis* (syn. *Glomus intraradices* BEG141) was maintained on leek. Leek roots with a mycorrhization rate of at least 85% (*M* value, Trouvelot et al., 1986) were thoroughly washed to remove any trace of soil, cut into 1 cm long pieces and used (50 mg FW/plant) to inoculate the mycorrhized (Myc) plants. Non-colonized leek roots were used to inoculate non-mycorrhized (NM) plants.

The plants were fertilized every second day with modified Long Ashton solution (Hewitt, 1966). Chlorinated oligo elements were used to get rid of sulfate sources, while MgSO4 was replaced by Mg(NO_3_)_2_. Final molarities in the nutrient solution were as follows: 127.1μM MnCl_2_, 11.7μM CuCl_2_ 2H_2_O, 14.7μM ZnCl_2_, 0.1μM Na_2_MoO_4_ 2H_2_O, 60μM FeNa EDTA, 0.5 mM H_3_BO_3_, 1 mM NaCl, 4 mM Ca(NO_3_)_3_ 4H_2_O, 7.99 mM KNO_3_, 1.5 mM Mg(NO_3_)_2_ 6H_2_O, 0.13 mM NaH_2_PO_4_ 2H_2_O. A 100 mM MgSO_4_ solution was added to reach 1μM (−S) and 1 mM (+S) SO^−2^_4_ final concentrations in the nutrient solutions. Up to 6 biological replicates were used for each condition and the experiment was repeated twice. All plants were harvested 4 weeks post inoculation (wpi), and their mycorrhization rates were assessed according to Vierheilig et al. (1998) and Trouvelot et al. (1986) before RNA extraction. Only plants exhibiting no AM fungal structure were used for the NM conditions, while fully colonized plants (*F* ≥ 90% and *M* ≥ 80%) were used as Myc replicates.

### RNA extraction

Leaf and root samples were collected, immediately frozen in liquid nitrogen and stored at −80°C. One hundred mg of frozen samples were ground in liquid nitrogen using the Trizol method (Invitrogen, Carlsbad, CA). Total RNAs were purified using the RNeasy Plant kit (Qiagen) according to the manufacturer's instructions. Genomic DNA was eliminated after treatment with DNase I for 20 min at 37°C using the DNA-free kit (Ambion, Austin, TX, USA). RNA was checked for purity and degradation by capillary electrophoresis using the Bio-Analyzer Experion (Bio-Rad; RNA Standard Sens kit; RNA StdSens chips). RNA concentrations were determined by spectrometry and only RNAs with an OD260:OD280 ratio ≥1.8 and no discernable degradation were used for the microarray experiments.

### Microarray design and oligo synthesis

*Medicago truncatula* genome have been annotated several times, since its sequencing, in order to better understand genes features and locations (http://jcvi.org for a track of the annotation history). The first annotation attempt (Mt1.0, 2005) was used to design the widely used Affymetrix GeneChip, which included several genes and/or EST contigs from *M. sativa* and a symbiotic bacteria. Due to the increasing discrepancies on genes ID and locations, observed during comparisons of results in different publications or between annotations (bioinformatics analyses conducted in the institute, data not shown), we decided to use the most recently available (Mt3.5) genome annotation (http://www.medicagohapmap.org).

The NimbleGen 12x135K design format (Nimblegen Systems, Inc., Madison, WI, USA) allowed for 4 distinct 60-mer oligos to be used for each of the 31,000 genes or so identified in Medicago genome. The microarray design, raw and processed data were uploaded into the Gene Expression Omnibus (GEO NCBI) public database (accession number GSE61357).

### cDNA synthesis, labeling, and hybridization

Double-Stranded cDNA (ds-cDNA) was synthesized from 10μg of total RNA using an Invitrogen SuperScript ds-cDNA synthesis kit in the presence of 250 ng of random hexamer primers. ds-cDNA was cleaned and labeled in accordance with the Nimblegen Gene Expression Analysis protocol (Nimblegen Systems, Inc., Madison, WI, USA). Briefly, ds-cDNA was incubated with 4μg of RNase A (Promega) at 37°C for 10 min and cleaned using 25:24:1 phenol:chloroform:isoamyl alcohol, followed by ice-cold absolute ethanol precipitation. For Cy3 labeling, a Nimblegen One-Color DNA labeling kit was used according to the manufacturer's guidelines detailed in the Gene Expression Analysis protocol (Nimblegen Systems, Inc., Madison, WI, USA). One μg of ds-cDNA was incubated for 10 min at 98°C with 2 OD of Cy3-9mer primer. Then, 100 pmol of deoxynucleoside triphosphates and 100U of the Klenow fragment (New England Biolabs, Ipswich, MA, USA) were added and the mix was incubated at 37°C for 2.5 h. The reaction was stopped by adding 0.1 volume of 0.5 M EDTA, and the labeled ds-cDNA was purified by isopropanol/ethanol precipitation.

Microarrays were hybridized at 38°C for 16–18 h with 6μg of Cy3-labeled ds-cDNA in Nimblegen hybridization buffer/hybridization component A in a hybridization chamber (Hybridization System—Nimblegen Systems, Inc., Madison, WI, USA). Following hybridization, washing was performed using the Nimblegen Wash Buffer kit (Nimblegen Systems, Inc., Madison, WI, USA).

### Data analysis

Slides were scanned at 5μm/pixel resolution using an Axon GenePix 4000 B scanner (Molecular Devices Corporation, Sunnyvale, CA, USA) piloted by GenePix Pro 6.0 software (Axon). Scanned images (TIFF format) were then imported into NimbleScan software (Nimblegen Systems, Inc., Madison, WI, USA) for grid alignment and expression data analyses. Expression data were normalized through quantile normalization and the Robust Multichip Average (RMA) algorithm included in the NimbleScan software.

Gene expression analysis was performed using the Array Star© software package from DNA Star (2014 DNASTAR, Inc.). A Student test was performed on the data with a false discovery rate (FDR) below 5%. Differentially expressed genes (DEGs) over different conditions were identified using a log2 (ratio) ≥1 and ≤1 filtering profile; the genes with a fold change ≤−2 and ≥2 compared to the relative control were retained.

At the time of our analysis, no database for direct association between gene IDs and gene ontology codes (GO) was available. For this reason, we used Blast2GO® software (Conesa et al., 2005; Götz et al., 2008) to create a database for the latest *Medicago truncatula* genome annotation (Mt3.5) including: annotation, GO IDs, KEGG and InterPro data (available upon request to the authors). To assess the relative enrichment of DEGs from each experimental condition compared to the whole genome, we created and tested individual files using Fisher's Exact test with Multiple Testing Correction of FDR (Benjamini and Hochberg). In addition, to visualize the gene expression profiles in different metabolic pathways we used MapMan software (Thimm et al., 2004) and KEGG Maps option in Blast2GO® software.

## Results

### *Medicago truncatula* genome

**Figure 1:**
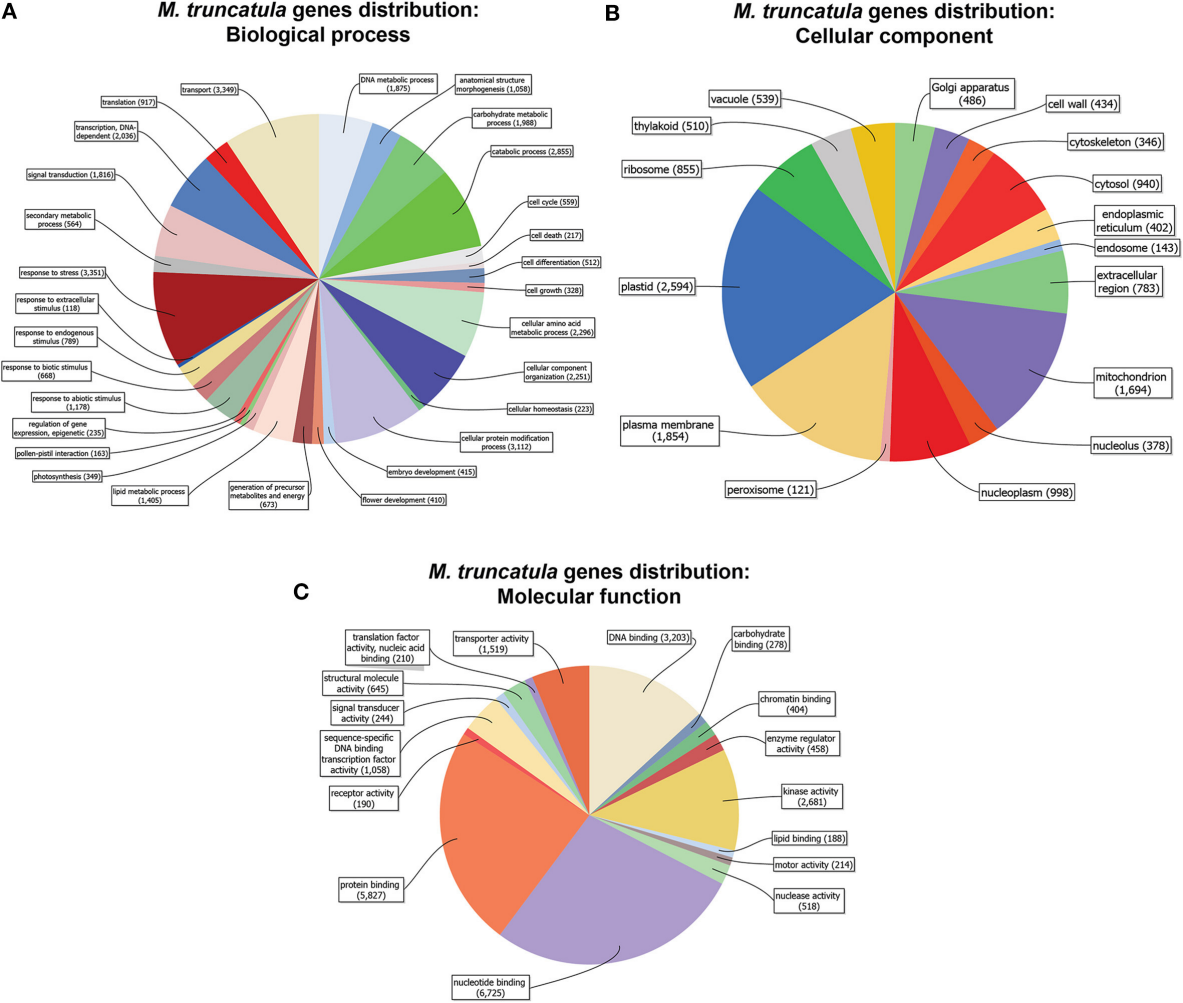
***Medicago truncatula* gene distribution (cutoff = 50 sequences)**. Genes are organized in categories according to biological process **(A)**, molecular function **(B)** and cellular component **(C)**, and represented by the amount of genes in each category.

Prior to investigating the transcriptional changes caused by S deficiency and/or mycorrhizal interaction in *Medicago truncatula*, we assessed its whole genome sequence distribution based on the “biological processes” or “molecular functions” categories (Figure 1). Most of the sequences coding for the 31,000 or so putative genes were associated to 2 or more GO IDs (up to 5) (Figures S1A,B), with a variable distribution among annotations according to the GO group (Biological process = P; Molecular function = F; Cellular component = C).

A vast majority of the genes had a putative function belonging to transport, stress response, protein modification, amino acid metabolism, and catabolic processes (Figure 1A). As far as cellular components are concerned, the genes were primarily associated to plastids, the plasma membrane, mitochondria, and the nucleoplasm (Figure 1B), while DNA- RNA- Protein-binding and kinase activities were the most abundant molecular functions (Figure 1C).

### Leaf transcriptional changes upon sulfur deficiency

**Figure 2:**
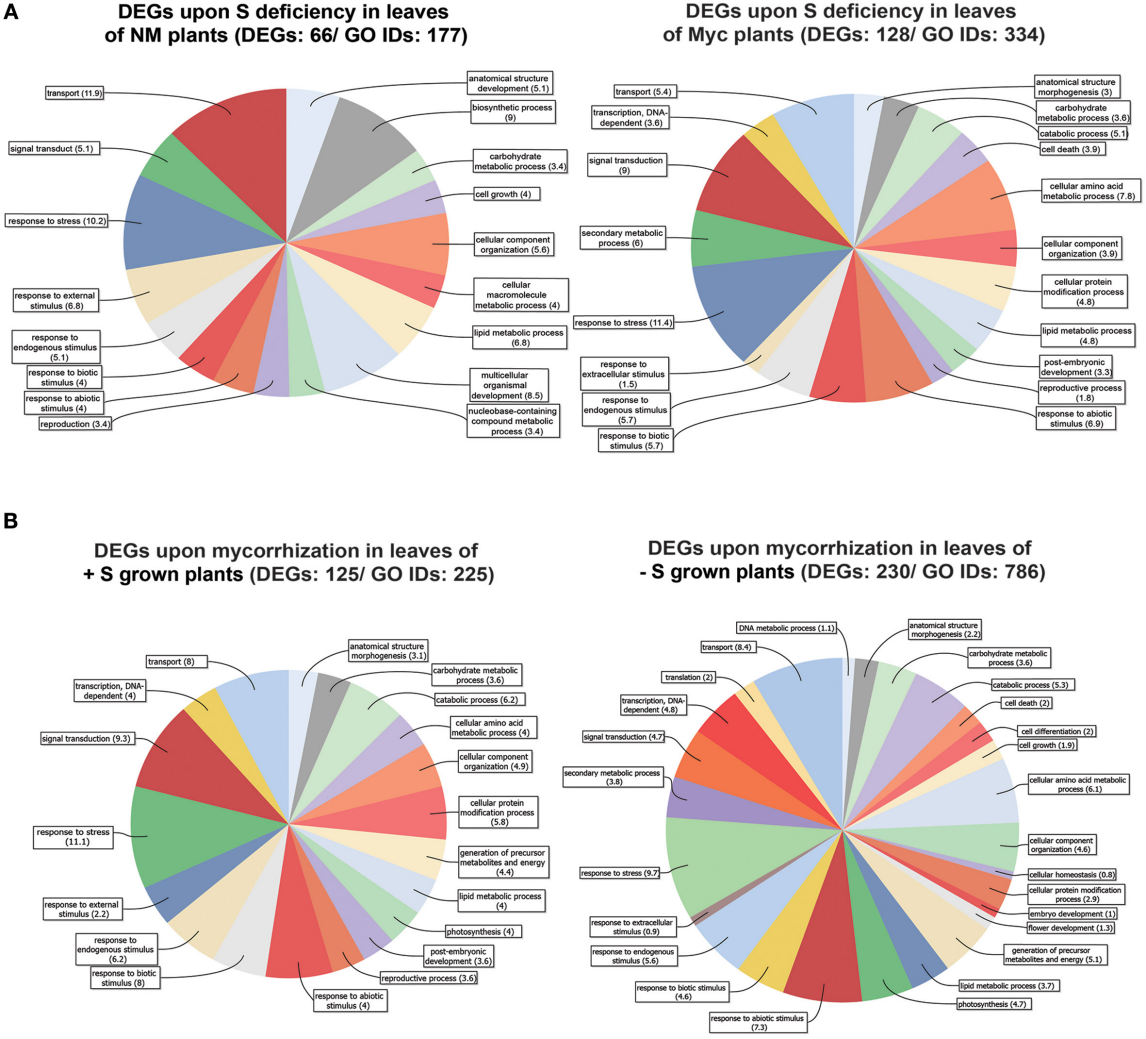
**Genes differentially expressed in *Medicago truncatula* leaves**. **(A)** Upon S deficiency stress in NM and Myc plants; **(B)** Upon mycorrhization in +S and −S conditions. Amount of DEGs and associated GO IDs are reported for each condition. Biological process categories are represented as percentage of the total GO identifiers.

We identified 66 differentially expressed genes (30 up- and 36 down-regulated) in NM plants upon S deficiency (Figure 2A, Figure S6A, Table S2). Six DEGs with transcription regulation activity (between 2.01 and 3.7 fold induction) and two Bowman-Birk-type proteinase inhibitors (2.1 and 2.2 fold) were among the up-regulated genes (Table S2). Ten genes with transporter activity (amino acid, oligopeptide, sulfate, copper and lipid) were among down-regulated (between −2.1 and −9.7 fold reduction), along with a cysteine synthase gene (−2.3) and a thiosulfate sulfur-transferase gene (−6.5).

Myc plants exhibited almost twice as many DEGs (128) upon S deficiency as NM ones (Table S2). This might explain the higher relative DEG abundance in some metabolic processes (Figure 2A, Figure S2) like the carbohydrate metabolism (2 fold), responses to abiotic (~3 fold), biotic (~3 fold) and endogenous (~2 fold) stimuli, signal transduction (~3 fold) and the stress response (~2 fold).

Comparing the distribution of differentially expressed genes between NM and Myc plants highlighted a different response in leaves of *M. truncatula* metabolic pathways according to symbiotic state. For instance the ascorbate-glutathione, lipids and phenylpropanoids pathways showed a different amount of DEGs (Figures S6A,B).

More in detail, only 8 out of 128 DEGs were up-regulated upon S deficiency in leaves of Myc plants, i.e., considerably less than in NM ones. Maybe due to the nutrient supply provided by the fungal symbiont, isoflavone synthase (IFS) Medtr7g027960_1, involved in the synthesis of key molecules in the phenylpropanoid biosynthetic pathway such as flavone, daidzeine and genistein, was up-regulated. Interestingly, several isoflavone-7-o-methyltransferase genes involved in the S-adenosyl-L-methionine (SAM) - S-adenosyl-L-homocysteine (SAH) cycling in the same pathway, were strongly down-regulated (Table S2) and possibly induced an accumulation of S-containing intermediate metabolites. Besides, transcript accumulation was reduced for several transcription factors and proteins with kinase activity.

A great number of DEGs identified in leaves of both NM and Myc plants were associated to biological processes such as transport, cellular organization, signal transduction and responses to stresses (Figures 2A, S2A, S6A,B). Among them 20 DEGs were found in both symbiotic conditions (Table 1). Interestingly, most of them were down-regulated, including several transcription factors, uncharacterized proteins and genes involved in S metabolism like the sulfate transporter Medtr5g061860.1 (MtSULTR2.1 from Casieri et al., 2012) and the thiosulfate sulfur-transferase Medtr8g075420.1. The only exceptions were three TIFY-domain-containing proteins, already described as putative transcription factors (Medtr5g013520.1, Medtr5g013530.1 and.2), which were up-regulated in Myc plants.

**Table 1:** **Genes differentially expressed upon S deficiency commonly found in the leaves of NM and Myc plants**.

**DEGs**	**NM fold change**	**Myc fold change**	**Annotation**	**InterProScan**
Medtr5g013530.2	3.68	−2.84	Protein tify	IPR010399 (SMART); IPR010399 (PFAM); IPR018467 (PFAM); IPR010399 (PROSITE_PROFILES)
Medtr5g013520.1	2.90	−2.11	Protein tify 10a	CYTOPLASMIC_DOMAIN (PHOBIUS); TRANSMEMBRANE (PHOBIUS); NON_CYTOPLASMIC_DOMAIN (PHOBIUS); TMhelix (TMHMM)
Medtr5g013530.1	2.77	−3.05	Protein tify 10a	IPR010399 (SMART); IPR010399 (PFAM); IPR018467 (PFAM); IPR010399 (PROSITE_PROFILES)
Medtr5g061860.1	−2.37	−2.12	Low affinity sulfate transporter 3-like	IPR011547 (PFAM); IPR002645 (G3DSA:3.30.750.GENE3D); IPR001902 (TIGRFAM); IPR002645 (PFAM); PF13792 (PFAM); PTHR11814:SF61 (PANTHER); PTHR11814 (PANTHER); IPR018045 (PROSITE_PATTERNS); NON_CYTOPLASMIC_DOMAIN (PHOBIUS); CYTOPLASMIC_DOMAIN (PHOBIUS); CYTOPLASMIC_DOMAIN (PHOBIUS); NON_CYTOPLASMIC_DOMAIN (PHOBIUS); TRANSMEMBRANE (PHOBIUS); NON_CYTOPLASMIC_DOMAIN (PHOBIUS); TRANSMEMBRANE (PHOBIUS); TRANSMEMBRANE (PHOBIUS); NON_CYTOPLASMIC_DOMAIN (PHOBIUS); CYTOPLASMIC_DOMAIN (PHOBIUS); NON_CYTOPLASMIC_DOMAIN (PHOBIUS); TRANSMEMBRANE (PHOBIUS); TRANSMEMBRANE (PHOBIUS); TRANSMEMBRANE (PHOBIUS); TRANSMEMBRANE (PHOBIUS); CYTOPLASMIC_DOMAIN (PHOBIUS); CYTOPLASMIC_DOMAIN (PHOBIUS); TRANSMEMBRANE (PHOBIUS); TRANSMEMBRANE (PHOBIUS); CYTOPLASMIC_DOMAIN (PHOBIUS); TRANSMEMBRANE (PHOBIUS); NON_CYTOPLASMIC_DOMAIN (PHOBIUS); TRANSMEMBRANE (PHOBIUS); TRANSMEMBRANE (PHOBIUS); TRANSMEMBRANE (PHOBIUS); CYTOPLASMIC_DOMAIN (PHOBIUS); NON_CYTOPLASMIC_DOMAIN (PHOBIUS); TRANSMEMBRANE (PHOBIUS); CYTOPLASMIC_DOMAIN (PHOBIUS); IPR002645 (PROSITE_PROFILES); IPR002645 (SUPERFAMILY); TMhelix (TMHMM)
Medtr3g109280.1	−2.41	−2.86	Hypothetical protein	IPR006747 (PFAM); PTHR31881 (PANTHER); PTHR31881:SF2 (PANTHER); CYTOPLASMIC_DOMAIN (PHOBIUS); TRANSMEMBRANE (PHOBIUS); NON_CYTOPLASMIC_DOMAIN (PHOBIUS); TRANSMEMBRANE (PHOBIUS); NON_CYTOPLASMIC_DOMAIN (PHOBIUS); TRANSMEMBRANE (PHOBIUS); CYTOPLASMIC_DOMAIN (PHOBIUS); CYTOPLASMIC_DOMAIN (PHOBIUS); TRANSMEMBRANE (PHOBIUS); TMhelix (TMHMM)
Medtr3g027090.1	−2.51	−2.26	Thioredoxin-like protein clot	IPR010357 (PFAM); IPR012336 (G3DSA:3.40.30.GENE3D); IPR010357 (PANTHER); IPR012336 (SUPERFAMILY)
Medtr4g087000.1	−2.90	−2.10	gibberellin 20 oxidase	no IPS match
Medtr4g086970.1	−3.06	−2.48	SENESCENCE-associated nodulin 1a	IPR002283 (PRINTS); IPR005123 (PFAM); IPR027443 (G3DSA:2.60.120.GENE3D); IPR026992 (PFAM); PTHR10209 (PANTHER); IPR005123 (PROSITE_PROFILES); SSF51197 (SUPERFAMILY)
Medtr1g015000.1	−3.40	−4.00	Copper transporter	IPR007274 (PFAM); IPR007274 (PANTHER); TRANSMEMBRANE (PHOBIUS); TRANSMEMBRANE (PHOBIUS); NON_CYTOPLASMIC_DOMAIN (PHOBIUS); CYTOPLASMIC_DOMAIN (PHOBIUS); NON_CYTOPLASMIC_DOMAIN (PHOBIUS); TRANSMEMBRANE (PHOBIUS); CYTOPLASMIC_DOMAIN (PHOBIUS); TMhelix (TMHMM)
Medtr4g086990.1	−3.51	−2.83	Gibberellin 20 oxidase	IPR002283 (PRINTS); IPR005123 (PFAM); IPR026992 (PFAM); IPR027443 (G3DSA:2.60.120.GENE3D); PTHR10209 (PANTHER); IPR005123 (PROSITE_PROFILES); SSF51197 (SUPERFAMILY)
Medtr4g015080.1	−3.94	−2.25	Peptide transporter ptr1	no IPS match
Medtr7g092240.1	−5.47	−4.11	Oligopeptide transporter opt family	no IPS match
Medtr4g132970.1	−5.50	−3.00	hypothetical protein	IPR006502 (TIGRFAM); IPR006502 (PFAM); PTHR31579 (PANTHER)
Medtr7g089780.1	−6.05	−3.22	Glycoside hydrolase family 31 protein	no IPS match
Medtr8g075420.1	−6.48	−5.52	Thiosulfate sulfurtransferase	IPR001763 (SMART); IPR001763 (G3DSA:3.40.250.GENE3D); IPR001763 (PFAM); PTHR13253 (PANTHER); IPR001763 (PROSITE_PROFILES); IPR001763 (SUPERFAMILY)
Medtr5g083770.1	−9.70	−6.48	Sodium-coupled neutral amino acid transporter	no IPS match
Medtr7g059010.1	−12.87	−6.09	hypothetical protein	Coil (COILS); Coil (COILS); IPR001440 (PFAM); IPR011990 (G3DSA:1.25.40.GENE3D); PTHR23083 (PANTHER); SSF48452 (SUPERFAMILY)
Medtr2g025520.1	−15.38	−3.65	Hypothetical protein	IPR006502 (TIGRFAM); IPR006502 (PFAM); PTHR31579:SF4 (PANTHER); PTHR31579 (PANTHER)
Medtr4g132940.1	−15.88	−5.91	Hypothetical protein	IPR006502 (TIGRFAM); IPR006502 (PFAM); PTHR31579 (PANTHER)
Medtr7g093010.1	−21.97	−10.25	myb family transcription factor apl	NON_CYTOPLASMIC_DOMAIN (PHOBIUS); CYTOPLASMIC_DOMAIN (PHOBIUS); TRANSMEMBRANE (PHOBIUS); TMhelix (TMHMM)

*Gene IDs, fold changes compared to +S conditions, putative annotations and associated InterProScan IDs are reported*.

At the cellular component level (Figure S2B), DEGs of Myc plants typically associated to the vacuole (11.3%) and the cell wall (13.2%) were not found in NM plants. Similarly, the molecular function distribution of the DEGs (Figure S2C) confirmed the differences between NM and Myc plants, with genes coding for receptor (8.1%), enzyme regulation (4.9%) and hydrolase activity (19.5%) exclusively found in S-Stressed Myc plants.

### Leaf transcriptional changes upon mycorrhization

In plants grown in +S conditions, 125 genes were differentially expressed upon mycorrhization (68 up- and 57 down-regulated), while 230 (57 up and 173 down) were differentially expressed in −S grown plants (Figure 2B, Figures S3A–C, Table S3).

Generally, the nutritional status of the plants affected leaf responses to mycorrhization by modifying the relative abundance of DEGs in cell wall, lipids and secondary metabolism pathways (Figures S7A,B). Several DEGs related to different biological processes exhibited different relative abundance levels in the two nutrient conditions, e.g., the genes associated to external stimulus responses were greatly affected in +S (4.3%) compared to −S conditions (0.9%). Similarly, several genes responsible for cellular protein modification were as much as twice in +S (5.6%) compared to −S (2.9%).

On the contrary, similar amounts of the genes associated to anatomical structure morphogenesis (3.1 and 2.2% in +S and −S), cellular component organization (4.9 and 4.6%), and carbohydrate metabolic processes (3.6%) were found.

We found 34 DEGs in common (14 up and 20 down) in the leaves of +S and −S plants upon AM interaction (Table 2). Some genes involved in the response to stress or biotic stimuli like the phospholipase D gene (Medtr1g083620.1, Medtr1g083620.2), the uroporphyrin methylase gene (Medtr7g046720.1), and a calmodulin-binding protein gene (Medtr3g083570.1), were up-regulated in both +S and −S conditions while some genes with transcription factor activity (Medtr5g013530.2, Medtr5g013520.1 and Medtr7g014570.2) were up-regulated in +S and down-regulated in −S conditions. Among the DEGs down-regulated in both nutritional conditions, we found four NADH-plastoquinone oxidoreductase genes (Medtr4g106580.1, Medtr5g032430.1, Medtr4g104220.1, Medtr3g008330.1), involved in ROS production, together with genes coding for chlorophyll A-B (Medtr5g098780.1, Medtr6g012080.1) and photosystems I and II (Medtr4g019000.1, Medtr7g017540.1, Medtr7g013040.1) binding proteins (Table 2).

**Table 2:** **Genes differentially expressed upon mycorrhization commonly found in the leaves of +S and −S grown plants**.

**DEGs**	**+S fold change**	**−S fold change**	**Annotation**	**InterProScan**
Medtr1g083620.1	3.65	2.12	Phospholipase d	IPR001736 (PFAM); IPR001849 (SMART); IPR011993 (G3DSA:2.30.29.GENE3D); IPR015679 (PANTHER); IPR016555 (PIR); IPR025202 (PFAM); G3DSA:3.30.870.10 (GENE3D), PTHR18896:SF2 (PANTHER), SSF50729 (SUPERFAMILY), SSF56024 (SUPERFAMILY)
Medtr1g083620.2	3.32	3.05	Phospholipase d	IPR015679 (PANTHER); PTHR18896:SF2 (PANTHER)
Medtr7g046720.1	3.12	4.09	Uroporphyrin methylase 1	IPR002213 (PANTHER); G3DSA:2.60.120.260 (GENE3D), G3DSA:3.40.50.2000 (GENE3D), PTHR11926:SF15 (PANTHER), SSF53756 (SUPERFAMILY)
Medtr3g026780.1	2.70	5.53	UDP-glucuronosyl/UDP-glucosyltransferas	IPR000878 (PFAM); IPR003043 (PROSITE); IPR006366 (TIGRFAMs); IPR014776 (G3DSA:3.30.950.GENE3D); IPR014777 (G3DSA:3.40.1010.GENE3D); PTHR21091 (PANTHER), PTHR21091:SF16 (PANTHER)
Medtr3g026820.1	2.62	3.34	Hypothetical protein	no IPS match
Medtr5g013530.2	2.49	−4.21	Protein tify	IPR010399 (PFAM); IPR018467 (PFAM)
Medtr4g015420.1	2.36	2.13	Glycoside hydrolase	IPR001360 (PRINTS); IPR013781 (G3DSA:3.20.20.GENE3D); IPR017853 (SUPERFAMILY); IPR018120 (PROSITE)
Medtr2g037790.1	2.28	3.38	Oxysterol-binding protein	IPR000648 (PANTHER); IPR018494 (PROSITE); SSF144000 (SUPERFAMILY)
Medtr7g118330.1	2.27	3.00	Protein late elongated hypocotyl	IPR001005 (PFAM); IPR006447 (TIGRFAMs); IPR009057 (G3DSA:1.10.10.GENE3D); IPR017930 (PROFILE); PTHR12802 (PANTHER), PTHR12802:SF23 (PANTHER)
Medtr5g013520.1	2.25	−2.73	Protein tify 10a	IPR010399 (PFAM); IPR018467 (PFAM)
AC2255195.1	2.16	2.20	Mlo-related protein	IPR004326 (PFAM); PTHR31942 (PANTHER), PTHR31942:SF0 (PANTHER)
Medtr3g083570.1	2.10	2.52	Calmodulin binding protein	PTHR31250 (PANTHER), PTHR31250:SF0 (PANTHER)
Medtr3g086830.1	2.02	−2.04	Hypothetical protein	no IPS match
Medtr7g014570.2	2.02	−2.00	Isoflavone-7-o-methyltransferase	IPR001077 (PFAM); G3DSA:3.40.50.150 (GENE3D), PTHR11746 (PANTHER), PTHR11746:SF53 (PANTHER), SSF53335 (SUPERFAMILY)
Medtr4g070120.1	−2.00	−3.99	nadh dehydrogenase subunit i	IPR000164 (PRINTS); IPR001450 (PFAM); IPR009072 (G3DSA:1.10.20.GENE3D); IPR012285 (G3DSA:1.10.1060.GENE3D); IPR017896 (PROFILE); IPR017900 (PROSITE); SSF54862 (SUPERFAMILY)
Medtr5g098780.1	−2.02	−2.15	Chlorophyll A-B binding protein	IPR001344 (PANTHER); IPR022796 (PFAM); IPR023329 (G3DSA:1.10.3460.GENE3D); PTHR21649:SF0 (PANTHER)
Medtr7g055910.1	−2.03	−2.50	Ribosomal protein s12	IPR005679 (PRINTS); IPR006032 (PANTHER); IPR012340 (G3DSA:2.40.50.GENE3D); IPR016027 (SUPERFAMILY)
Medtr4g072590.1	−2.03	−3.22	Auxin-induced protein	IPR003676 (PFAM); PTHR31929 (PANTHER)
Medtr8g020640.1	−2.04	−2.44	Serine threonine kinase	IPR000719 (PROFILE); IPR001245 (PFAM); IPR008271 (PROSITE); IPR011009 (SUPERFAMILY); IPR017441 (PROSITE); G3DSA:1.10.510.10 (GENE3D), G3DSA:3.30.200.20 (GENE3D), PTHR24420 (PANTHER), PTHR24420:SF191 (PANTHER)
Medtr4g106580.1	−2.08	−2.03	Nadh-plastoquinone oxidoreductase subunit 4	PTHR22773 (PANTHER), PTHR22773:SF61 (PANTHER)
Medtr5g032430.1	−2.13	−3.93	Nadh-plastoquinone Oxidoreductase subunit 4	IPR001750 (PFAM); IPR003918 (PRINTS); PTHR22773 (PANTHER), PTHR22773:SF61 (PANTHER)
Medtr6g012080.1	−2.14	−2.02	Chlorophyll A-B binding protein	IPR001344 (PANTHER); IPR022796 (PFAM); IPR023329 (G3DSA:1.10.3460.GENE3D); PTHR21649:SF0 (PANTHER)
Medtr7g112660.1	−2.16	−2.27	Magnesium proton exchanger	no IPS match
Medtr4g098010.1	−2.17	−2.09	Cullin-like protein1	no IPS match
Medtr4g019000.1	−2.19	−2.60	Photosystem ii protein i	IPR003686 (PFAM); IPR003687 (PRODOM)
AC23366246.1	−2.21	−5.34	Rna polymerase beta subunit	no IPS match
Medtr4g104220.1	−2.23	−2.41	Nadh-plastoquinone oxidoreductase subunit j	IPR001268 (PRODOM); IPR020396 (PROSITE); PTHR10884 (PANTHER), PTHR10884:SF1 (PANTHER), SSF143243 (SUPERFAMILY)
Medtr3g008330.1	−2.27	−2.82	nadh dehydrogenase subunit 4	IPR001750 (PFAM); PTHR22773 (PANTHER), PTHR22773:SF61 (PANTHER)
Medtr7g118030.1	−2.32	−2.04	Hypothetical protein	no IPS match
Medtr8g073850.1	−2.44	−2.25	Disease resistance response protein	IPR004265 (PFAM)
Medtr7g017540.1	−2.45	−2.18	Photosystem ii protein d1	IPR000484 (G3DSA:1.20.85.GENE3D)
Medtr7g013040.1	−2.48	−2.83	Photosystem i apoprotein a1	IPR001280 (G3DSA:1.20.1130.GENE3D); SSF81558 (SUPERFAMILY)
Medtr8g106380.1	−2.54	−2.69	Hypothetical protein	no IPS match
Medtr3g007900.1	−2.57	−2.10	Gibberellin induced protein	IPR003854 (PFAM)

*Gene IDs, fold changes compared to NM plants, putative annotations and associated InterProScan IDs are reported*.

Similar abundances of DEGs associated to the stress response (10.7 and 9.7% in +S and −S conditions, respectively), transport processes (7.7 and 8.4%), carbohydrate (3.4 and 3.6%) and lipid metabolic process (3.9 and 3.7%) were observed (Figure S3A).

### Root transcriptional changes upon sulfur deficiency

**Figure 3:**
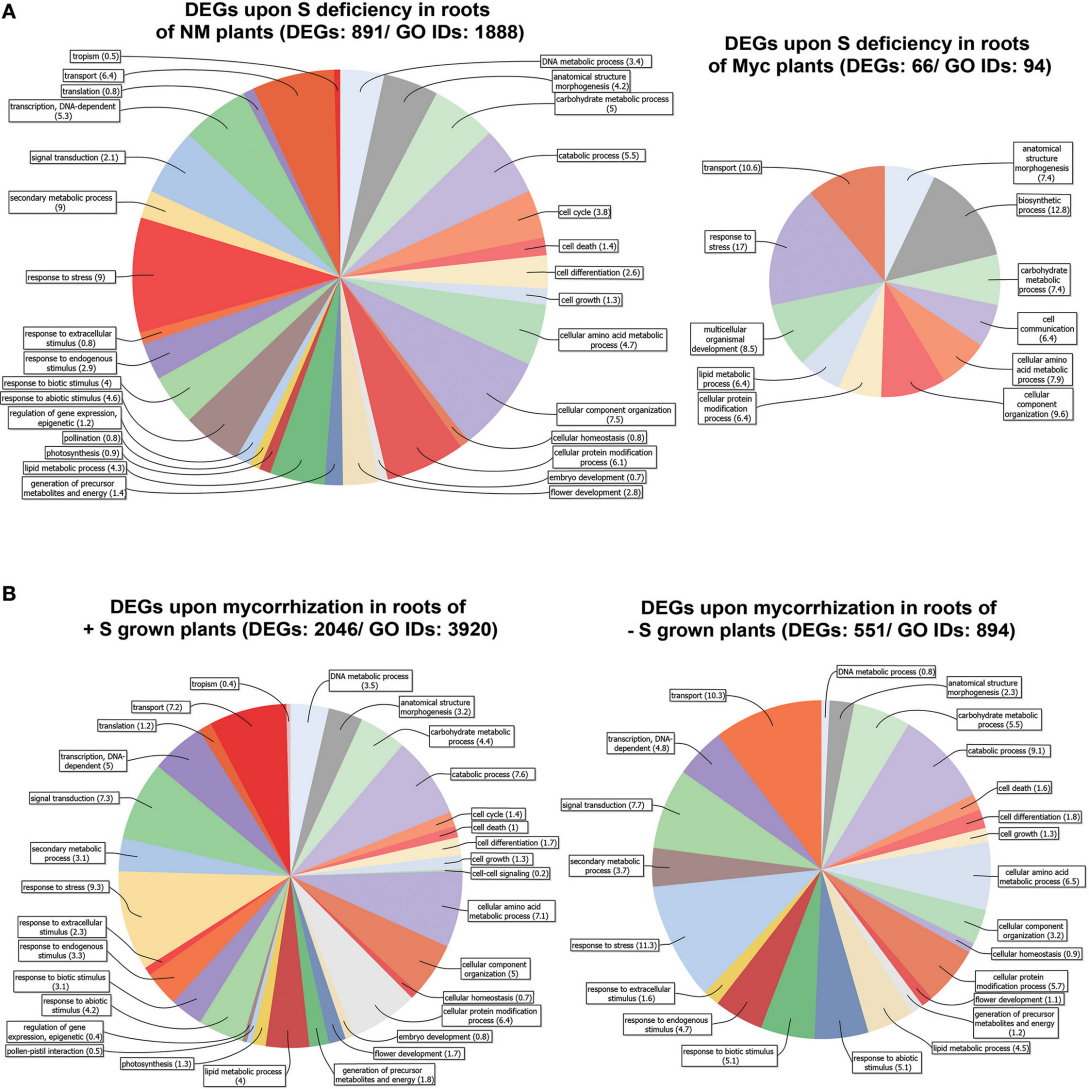
**Genes differentially expressed in *Medicago truncatula* roots**. **(A)** Upon S deficiency stress in NM and Myc plants; **(B)** Upon mycorrhization in +S and −S conditions. Amount of DEGs and associated GO IDs are reported for each condition. Biological process categories are represented as percentage of the total GO identifiers.

The roots of NM plants responded to S deficiency by changing the expression profile of a great number of genes (891), 13.5 times more than in Myc plants (66) (Figure 3A, Figures S6C,D, Table S4). The vast majority of DEGs found in NM roots (695 out of 891) were up-regulated upon S deficiency (Table S4). The metabolic pathways best represented by the DEGs were the starch, sucrose, purine, pentose/glucuronate interconversion and thiamine metabolisms, accounting for 73 (10.4%) up-regulated genes coding for 28 different enzymes. Nevertheless, genes involved in the metabolism of pyrimidine (8 genes, 4 enzymes), amino/nucleotide sugars (7 genes, 7 enzymes), glutathione (7 genes, 6 enzymes), glycerolipid and riboflavin (7 genes, 4 enzymes) were also up-regulated. Among the most severely down-regulated DEGs, genes coding for several transporters (peptide, copper, and amino acid) and oxidoreductases (quinone and gibberellin) exhibited up to −61.4 fold reduction in transcript accumulation.

S deficiency affected gene expression in the root tissues of Myc plants, with 28 out of 66 DEGs up regulated. Among them, several genes coding for kinases and receptor-like kinases exhibited increased transcript accumulation up to 3.04 fold compared to NM S-Stressed tissues, together with 9 peroxidases and two pectinesterase inhibitors (Table S4). Among the most severely down-regulated genes were those coding for the myb transcription factor Medtr7g093010.1 (−25.1 fold), for glycoside hydrolase Medtr7g089780.1 (−16 fold) and for gibberellin-20-oxidase Medtr4g086990.1 (−13.4 fold).

According to symbiotic state of the plants several metabolic pathways were characterized by a different DEGs distribution upon S deficiency. For instance, Ascorbate-Glutathione, light reactions, cell wall, lipids and several secondary metabolism pathways showed higher number of DEGs in NM plants compared to Myc ones (Figures S6C,D). Besides the fact that such differences in DEG amounts could provide clues for a more complete representation of the biological processes and cellular components involved (Figures S4A,B), several of these processes displayed increased relative DEG abundance in symbiotic conditions. For instance, the DEGs associated to response to stress accounted for 9 and 17.2% of the total DEGs in NM and Myc plants, respectively. The tendency was similar for other processes such as transport processes (6.44 and 11.1%), anatomical structure morphogenesis (4.3 and 7.1%), carbohydrate (5.1 and 7.1%), amino acid (4.7 and 6.1%) and lipid metabolism (4.3 and 6.1%).

Most of the DEGs identified upon S deficiency exhibited nucleotide-binding activity (26 and 35.7%) and kinase activity (9.1 and 16.7%). The DEGs associated with transporter activity represented 16.7% of total DEGs in Myc plants, almost twice as many as in NM ones (7.5%). Moreover, as observed in leaf tissues, Myc plants responded to S deficiency with a consistent amount of DEGs associated to hydrolase activity, with 30.9% of total DEGs in the roots and 19.5% in the leaves (Figures S2C, S4C).

Thirty-Six genes were differentially expressed upon S deficiency (5 up- and 31 down-regulated) in the roots of both NM and Myc plants (Table 3). Interestingly, four genes involved in transporter activity were down-regulated, including a lysine-histidine transporter (Medtr5g054730.1), an oligopeptide (Medtr7g092240.1), a copper (Medtr1g015000.1) and a Na-coupled amino acid transporter (Medtr5g083770.1), while a proton-dependent oligopeptide transporter (Medtr5g055000.2) was up-regulated. Similarly, some genes involved in sulfur-transferase activity were regulated contrastingly. For instance, the gene coding for a flavonol sulfur-transferase-like protein (Medtr8g040250.1) was upregulated, while the genes coding for a thiosulfate (Medtr8g075420.1) and a glutathione sulfur-transferase (Medtr7g100320.1) were down-regulated in both NM and Myc plants.

**Table 3:** **Genes differentially expressed upon S deficiency commonly found in the roots of NM and Myc plants**.

**DEGs**	**NM fold**	**Myc fold**	**Annotation**	**InterProScan**
	**change**	**change**		
Medtr5g055000.2	6.79	2.56	Proton-dependent oligopeptide transporter (POT)	IPR000109 (PANTHER); IPR016196 (SUPERFAMILY); G3DSA:1.20.1250.20 (GENE3D), PTHR11654:SF57 (PANTHER), tmhmm (TMHMM)
Medtr8g040250.1	2.72	3.27	Flavonol sulfotransferase-like protein	IPR000863 (PFAM); G3DSA:3.40.50.300 (GENE3D), PTHR11783 (PANTHER), PTHR11783:SF1 (PANTHER), SSF52540 (SUPERFAMILY)
AC22550748.1	2.59	2.19	Phospholipase d epsilon	IPR001736 (PFAM); IPR008973 (SUPERFAMILY); IPR011402 (PIR); IPR015679 (PANTHER); IPR024632 (PFAM); G3DSA:2.60.40.150 (GENE3D), G3DSA:3.30.870.10 (GENE3D), PTHR18896:SF30 (PANTHER), SSF56024 (SUPERFAMILY)
AC23366039.1	2.39	2.57	Glycoside hydrolase	IPR000322 (PANTHER); IPR011013 (SUPERFAMILY); PTHR22762:SF9 (PANTHER), SignalP-NN(euk) (SIGNALP), tmhmm (TMHMM)
Medtr3g010780.1	2.35	2.26	Pectinesterase inhibitor	IPR000070 (PFAM); IPR006501 (G3DSA:1.20.140.GENE3D); IPR011050 (SUPERFAMILY); IPR012334 (G3DSA:2.160.20.GENE3D); IPR018040 (PROSITE); PTHR31707 (PANTHER), PTHR31707:SF0 (PANTHER)
Medtr5g006710.1	−3.32	−2.79	12-oxophytodienoate reductase	IPR001155 (PFAM); IPR013785 (G3DSA:3.20.20.GENE3D); PTHR22893 (PANTHER), PTHR22893:SF13 (PANTHER), SSF51395 (SUPERFAMILY)
Medtr3g073470.1	−3.43	−2.33	Serine hydroxymethyltransferase	IPR001085 (PANTHER); IPR015421 (G3DSA:3.40.640.GENE3D); IPR015422 (G3DSA:3.90.1150.GENE3D); IPR015424 (SUPERFAMILY); IPR019798 (PROSITE); PTHR11680:SF0 (PANTHER)
Medtr8g075420.1	−3.52	−2.39	Thiosulfate sulfurtransferase	IPR001763 (G3DSA:3.40.250.GENE3D); PTHR13253 (PANTHER), PTHR13253:SF25 (PANTHER)
Medtr7g058930.1	−3.64	−2.37	Tetratricopeptide TPR1	IPR001440 (PFAM); IPR011990 (G3DSA:1.25.40.GENE3D); IPR019734 (PROFILE); PTHR23083 (PANTHER), PTHR23083:SF190 (PANTHER), SSF48452 (SUPERFAMILY)
Medtr7g100320.1	−3.79	−2.13	Glutathione S-transferase	IPR004045 (PROFILE); IPR010987 (G3DSA:1.20.1050.GENE3D); IPR012336 (G3DSA:3.40.30.GENE3D); IPR017933 (PROFILE); PTHR11260 (PANTHER), PTHR11260:SF14 (PANTHER), PF13410 (PFAM), PF13417 (PFAM)
Medtr5g054730.1	−4.15	−2.41	Lysine histidine transporter	IPR013057 (PFAM); PTHR22950 (PANTHER), PTHR22950:SF9 (PANTHER)
Medtr4g086980.1	−4.73	−2.60	gibberellin 20 oxidase	IPR002283 (PRINTS); IPR005123 (PFAM); IPR026992 (PFAM); G3DSA:2.60.120.330 (GENE3D), PTHR10209 (PANTHER), PTHR10209:SF55 (PANTHER), SSF51197 (SUPERFAMILY)
Medtr5g010410.1	−4.93	−2.75	Heavy metal p-type atpase	IPR001757 (PRINTS); IPR005834 (PFAM); IPR023214 (G3DSA:3.40.50.GENE3D); IPR023299 (G3DSA:3.40.1110.GENE3D); PTHR24093 (PANTHER), PTHR24093:SF42 (PANTHER)
Medtr6g012140.1	−5.65	−2.52	Quinone oxidoreductase-like protein	IPR002085 (PANTHER); IPR016040 (G3DSA:3.40.50.GENE3D); PF13602 (PFAM), SSF51735 (SUPERFAMILY)
Medtr1g079520.1	−5.92	−3.05	Protein kinase	IPR000719 (PFAM); IPR001611 (PFAM); IPR002885 (PFAM); IPR003034 (G3DSA:1.10.720.GENE3D); IPR008271 (PROSITE); IPR011009 (SUPERFAMILY); IPR013210 (PFAM); IPR017441 (PROSITE); G3DSA:1.10.510.10 (GENE3D), G3DSA:3.80.10.10 (GENE3D), PTHR24420 (PANTHER), PTHR24420:SF134 (PANTHER), PF13516 (PFAM), PF13855 (PFAM), SSF52047 (SUPERFAMILY), SSF52058 (SUPERFAMILY), SSF68906 (SUPERFAMILY)
Medtr5g010420.1	−6.72	−2.46	Heavy metal p-type atpase	IPR001757 (TIGRFAMs); IPR006121 (PFAM); IPR006122 (TIGRFAMs); IPR006403 (PRINTS); IPR008250 (PFAM); IPR017969 (PROSITE); IPR018303 (PROSITE); IPR023214 (G3DSA:3.40.50.GENE3D); IPR023300 (G3DSA:2.70.150.GENE3D); G3DSA:3.30.70.100 (GENE3D), PTHR24093 (PANTHER), PTHR24093:SF42 (PANTHER), SSF81653 (SUPERFAMILY), SSF81665 (SUPERFAMILY)
Medtr1g081610.1	−6.90	−2.34	Cysteine synthase	IPR001216 (PROSITE); IPR001926 (PFAM); IPR005856 (TIGRFAMs); IPR005859 (TIGRFAMs); G3DSA:3.40.50.1100 (GENE3D), PTHR10314 (PANTHER), PTHR10314:SF8 (PANTHER)
Medtr4g005810.1	−7.82	−3.16	f-box lrr-repeat protein	G3DSA:3.80.10.10 (GENE3D), PTHR23125 (PANTHER), SSF52047 (SUPERFAMILY)
Medtr4g087000.1	−8.91	−4.29	Gibberellin 20 oxidase	IPR002283 (PRINTS); IPR005123 (PFAM); IPR026992 (PFAM); G3DSA:2.60.120.330 (GENE3D), PTHR10209 (PANTHER), PTHR10209:SF55 (PANTHER), SSF51197 (SUPERFAMILY)
Medtr7g092240.1	−9.36	−4.20	Oligopeptide transporter opt family	IPR004648 (TIGRFAMs); IPR004813 (PFAM); PTHR22601 (PANTHER)
Medtr5g054300.1	−11.19	−2.15	SANT/Myb domain	IPR001005 (PFAM); IPR006447 (TIGRFAMs); IPR009057 (G3DSA:1.10.10.GENE3D); IPR017930 (PROFILE); PTHR31003 (PANTHER)
Medtr3g109280.1	−11.35	−3.65	Unknown protein	IPR006747 (PFAM); PTHR31881 (PANTHER)
Medtr6g012100.1	−11.55	−3.08	Quinone oxidoreductase-like protein	PF13602 (PFAM)
Medtr4g086990.1	−17.83	−13.41	Gibberellin 20 oxidase	IPR002283 (PRINTS); IPR005123 (PFAM); IPR026992 (PFAM); G3DSA:2.60.120.330 (GENE3D), PTHR10209 (PANTHER), PTHR10209:SF55 (PANTHER), SSF51197 (SUPERFAMILY)
Medtr4g132970.1	−19.54	−3.32	Unknown protein	IPR006502 (TIGRFAM); IPR006502 (PFAM); PTHR31579 (PANTHER)
Medtr1g015000.1	−19.88	−4.12	Copper transporter	IPR007274 (PANTHER); PTHR12483:SF1 (PANTHER)
Medtr4g086970.1	−20.28	−7.03	Senescence-associated nodulin 1a	IPR002283 (PRINTS); IPR005123 (PFAM); IPR026992 (PFAM); G3DSA:2.60.120.330 (GENE3D), PTHR10209 (PANTHER), PTHR10209:SF55 (PANTHER), SSF51197 (SUPERFAMILY)
Medtr6g012170.1	−32.25	−5.25	Quinone oxidoreductase-like protein	IPR002085 (PANTHER); IPR002364 (PROSITE); IPR011032 (SUPERFAMILY); IPR013154 (PFAM); IPR016040 (G3DSA:3.40.50.GENE3D); G3DSA:3.90.180.10 (GENE3D), PTHR11695:SF34 (PANTHER), PF13602 (PFAM), SSF51735 (SUPERFAMILY)
Medtr6g012160.1	−38.03	−6.31	Quinone oxidoreductase-like protein	IPR002085 (PANTHER); IPR002364 (PROSITE); IPR011032 (SUPERFAMILY); IPR013149 (PFAM); IPR013154 (PFAM); IPR016040 (G3DSA:3.40.50.GENE3D); G3DSA:3.90.180.10 (GENE3D), PTHR11695:SF34 (PANTHER), SSF51735 (SUPERFAMILY)
Medtr5g083770.1	−40.53	−10.71	Sodium-coupled neutral amino acid transporter	IPR013057 (PFAM); PTHR22950 (PANTHER)
Medtr4g132940.1	−41.06	−4.51	Protein	IPR006502 (TIGRFAM); IPR006502 (PFAM); PTHR31579 (PANTHER)
Medtr7g093010.1	−43.15	−25.14	myb family transcription factor apl	IPR001005 (PFAM); IPR006447 (TIGRFAMs); IPR009057 (G3DSA:1.10.10.GENE3D); PTHR31314 (PANTHER)
Medtr7g089780.1	−46.07	−16.02	Glycoside hydrolase family 31 protein	IPR000322 (PANTHER); IPR011013 (SUPERFAMILY); IPR017853 (SUPERFAMILY); PTHR22762:SF5 (PANTHER), SSF51011 (SUPERFAMILY)
Medtr8g042370.1	−47.19	−10.79	Serine-threonine protein plant-	IPR000719 (PROFILE); IPR001245 (PFAM); IPR008266 (PROSITE); IPR011009 (SUPERFAMILY); IPR014729 (G3DSA:3.40.50.GENE3D); G3DSA:1.10.510.10 (GENE3D), G3DSA:3.30.200.20 (GENE3D), PTHR24420 (PANTHER)
Medtr7g059010.1	−49.75	−12.24	Tetratricopeptide TPR1	IPR001440 (PFAM); IPR011990 (G3DSA:1.25.40.GENE3D); PTHR23083 (PANTHER), PTHR23083:SF190 (PANTHER), SSF48452 (SUPERFAMILY)
Medtr2g025520.1	−61.44	−6.32	Unknown protein	IPR006502 (TIGRFAM); IPR006502 (PFAM); PTHR31579 (PANTHER)

*Gene IDs, fold changes compared to +S conditions, putative annotations and associated InterProScan IDs are reported*.

Genes coding for gibberellin oxidases (Medtr4g086980.1, Medtr4g087000.1, and Medtr4g086990.1), quinone oxidoreductase (Medtr6g012140.1, Medtr6g012100.1, Medtr6g012170.1, and Medtr6g012160.1) and transcription factors (Medtr5g054300.1, Medtr7g093010.1 and Medtr7g059010.1) were also down-regulated upon S deficiency in both NM and Myc conditions.

### Root transcriptional changes upon mycorrhization

Among the different conditions we tested, the root tissues of +S plants exhibited the highest amount of DEGs upon mycorrhization (Figure 3B, Figures S5, S7C). Nearly half (1230) of the DEGs identified in the roots of +S plants were up-regulated (Table S5). The genes most affected by the symbiosis with the AM fungus coded for glutathione S-transferases (7 genes up-regulated), proteinases (13 up, 3 down), lectins (21 up), lipases (21 up, 3 down), inhibitors (29 up, 5 down), transcription factors (67 up, 19 down) and transporters (46 up, 20 down).

In the root tissues of −S plants, 551 genes displayed a significantly different transcript accumulation (491 up- and 60 down-regulated). Genes coding for glutathione S-transferases (3 up), proteinases (9 up), lectins (15 up), lipases (5 up, 1 down), inhibitors (16 up, 1 down), transcription factors (31 up, 3 down) and several transporters (28 up, 2 down) were also affected by the AM interaction (Table S5).

Although several biological processes were affected by the AM interaction in both +S and −S conditions, we observed only slight differences in DEG relative abundance levels (Figures S5A, S7C,D). We could note the slightly increased numbers of DEGs involved in the response to biotic stimuli (5.9 and 8.3% in +S and −S conditions) and the decreased amounts of those related to DNA (6.8 and 1.3%) and amino acid (13.6 and 10.5%) metabolic processes.

The DEGs associated to vacuole, plastid, and plasma membrane cellular components represented the majority of genes differentially expressed upon mycorrhization in the roots of +S and −S grown plants (Figure S5B). Further analysis of DEG distribution highlighted the nucleotide binding, protein binding and transporter functions as the most affected by mycorrhization in both nutrient conditions (Figure S5C). Besides, the most evident differences were for those DEGs involved in transport activity: they represented 6.8 and 11.3% of total DEGs in +S and −S conditions, respectively.

Noteworthy, the roots of +S and −S plants shared a great number of DEGs (424) upon mycorrhization. Most of them (403 genes, 95%) were up-regulated (Table S1).

## Discussion

### Effects of sulfur deficiency

In our experimental conditions, the effect of S deficiency in NM plants was obvious, with reduced leaf and roots biomasses, shorter branches of vegetative organs and fewer leaves (data not shown). These results are in accordance with other reports on the effects of S starvation on Medicago and other crop plants (McGrath et al., 1996; Casieri et al., 2012). They can be explained by a decrease in S-containing metabolites (e.g., O-acetyl-L-serine, cysteine, glutathione and S-adenosyl-methionine) that directly affects plant development (Ohkama-Ohtsu and Wasaki, 2010), or by the effect of the induction of 12-oxophitodienoate reductase and ACC synthase, respectively involved in jasmonate and ethylene biosynthesis (D'Hooghe et al., 2013).

Iqbal et al. (2013) have recently reviewed the crass-talk between S assimilation and ethylene signaling in different plant species. As a general effect of S withdrawal from the growing medium reduced levels of sulfate, cysteine and glutathione are expected, leading to the induction of transcriptional changes (Maruyama-Nakashita et al., 2003; Nikiforova et al., 2003). In accordance with this, we observed several transporter, cysteine, and glutathione synthase genes affected by S deficiency in both leaves and roots. By contrast, O-acetylserine accumulate in *A. thaliana* tissues when plants are facing S starvation (Kopriva et al., 2009). O-acetylserine most probably has a regulatory role in the pathway leading to cysteine synthase and its levels might acts as transcriptional regulator during S starvation (Koprivova et al., 2000; Ohkama et al., 2002).

More in detail, genes associated to regulation and transport mechanisms were the most affected by S deficiency in the leaves of NM plants. The down-regulation of genes involved in amino acid, peptide, sulfate and lipid transport, can be explained by the effects of reduced S availability on the biosynthesis of amino acids, proteins and sulfolipids. Our data are in accordance with previous works on soybean (*Glycine max*) by Sunarpi and Anderson (1997a,b), who reported that the remobilization of S compounds stored in leaves in case of S deficiency is affected by N availability. Low N nutrition promoted the loss of S from mature leaves to the benefit of the developing ones, while high levels of N inhibited this process. Dubousset et al. (2009) confirmed this interconnection between S and N availability in oilseed rape (*Brassica napus*) and observed that the recycling of endogenous foliar S compounds may occur without any acceleration of the leaf senescence process provided that N remains a non-limiting nutrient.

In our experiments N was not a limiting element, so that NM plants were probably harvested before showing any sign of senescence. Medicago plants apparently cope with a lack of amino acids and sulfolipids needed to build new tissues by strongly reducing their growth rate. The up-regulation of proteinase inhibitors to reduce the recycling of S compounds from older tissues could support this hypothesis.

In the root tissues of NM plants several transporters and quinone/gibberellin oxido-reductases were down-regulated, and more generally carbohydrate and purine metabolism pathways were the most affected. These data suggest that to adapt to S deficiency, the plant represses the pathways involved in sugar translocation and tissue growth by means of a fine regulation of cellular responses. Indeed, several genes coding for transcription factors (32) and proteins with receptor-kinase activity (25) were up-regulated.

Although the AM interaction increased plant S availability, transcript accumulation in the leaves of Myc plants indicated that several biosynthesis pathways were affected by S deficiency and mostly down-regulated. In particular, gene regulation of the phenylpropanoid pathway seems to suggest a preferential accumulation of S-containing intermediate metabolites to be further reallocated within the plant tissues (Figure S6). Similarly, Davey et al. (2004) observed that phenylpropanoid concentrations in leaves of *Plantago lanceolata* were also altered by changes in resource availability.

In the root tissues of Myc plants, contrasting with NM ones, we identified several genes up-regulated upon S deficiency associated with transporter activity. This might be due to the increased reallocation needs related to the nutrient exchanges with the mycobiont. Similarly, kinases and receptor-like kinases displayed increased transcript accumulation.

The common DEGs in NM and Myc plants might represent the basal gene regulation of Medicago plants during S starvation, despite their interaction with the fungal symbiont. Only 20 DEGs, mostly transporters and transcription factors, were down-regulated in the leaves of both NM and Myc plants. Similarly, in root tissues the basal plant response to S deficiency seems to have mostly resulted in the down-regulation of several genes, among which genes coding for thiosulfate and glutathione sulfurtransferases, gibberellin oxidases and quinone oxidoreductases. According to Lo et al. (2008), who used rice (*Oryza sativa*) mutant lines of closely related gibberellin oxidases, these genes play a role in the root system development. Moreover, quinone oxidoreductases (EC 1.6.5) can catalyze quinone redox changes, possibly affecting the capability of root tissues to coordinate symbiotic/pathogenic interactions (Siqueira et al., 1991; Hirsch et al., 2002) and to overcome the effects of ROS.

### Effects of the mycorrhizal interaction

To investigate whether the plant nutritional state plays a role during the establishment of a functional AM interaction, the transcriptomic data of our experiments were mined to better understand the plants' responses to mycorrhizal interaction in +S and −S conditions.

In *M. truncatula* leaves, we identified several genes differentially expressed upon AM interaction in +S (125) and −S (230) grown plants; among them 34 (27.2 and 14.8% of total DEGs in +S and −S) were differentially expressed in both conditions.

Genes associated to stress (biotic and abiotic) responses and transporter-related genes were better represented in the leaves of −S plants. These data suggest how the AM interaction helps plants respond to difficult developmental conditions such as S deficiency at the transcriptomic level. Our results are in agreement with previous reports on the transporter activity of Myc plants grown in different S conditions (Casieri et al., 2012). The higher amount of affected transporters strongly highlights the increased reallocation needs of plants in −S conditions upon mycorrhization, and indirectly confirms a more important nutrient contribution of the mycobiont compared to +S conditions.

Two genes coding for a phospholipase D (PLD) and a calmodulin (CaM-) binding protein were up-regulated. These two genes might have a key role in regulating different plant processes and stress responses. In fact, PLDs are mainly involved in responses to abiotic and biotic stresses, plant development and seed quality. Their activation regulates the production of the lipid messenger phosphatidic acid (PA), and the selective hydrolysis of membrane lipids (Munnik, 2001; Bargmann and Munnik, 2006; Zhang et al., 2009). CaM-binding proteins, *via* a signaling cascade mechanism, mainly react to biotic and abiotic stresses to regulate the activity of numerous proteins with diverse cellular functions (Bouché et al., 2005).

Interestingly, the capability of plants to regulate ROS production through NADH/plastoquinone oxidoreductases, the synthesis of chlorophyll A-B photosystem-binding proteins seems to be negatively affected by the AM interaction.

Due to the increased sink strength upon AM interaction, the plant is thought to increase its photosynthate production. In *M. truncatula*, the role of sugar transporters (Kaschuk et al., 2009; Doidy et al., 2012; Casieri et al., 2013) as well as the efficiency and capacity of the photosynthetic apparatus (Rehman, 2010) support this hypothesis. Interestingly, our results point in the opposite direction, leaving the question open as to other mechanisms of sugar production for plant growth and of nutrient exchanges with the mycobiont.

We identified the highest amounts of DEGs in the roots of +S (2046) and −S Myc plants (551). Although the total amount in −S conditions was significantly lower (1/4) compared to +S, their relative distributions were similar and many genes were common to +S and −S conditions. Similar amounts of DEGs were reported in previous works, where the *M. truncatula* transcriptome was assessed during AM interactions (Manthey et al., 2004; Hogekamp et al., 2011).

Among these DEGs the expression profiles of several mycorrhizal related ones confirmed previous studies based on transcriptomic and functional approaches. Eight members of the blue copper protein family were strongly up-regulated upon mycorrhization in both +S and −S conditions (Table S5), together with the phosphate transporter MtPT4 (Medtr5g068140.1; Harrison et al., 2002; Javot et al., 2011). This results confirms previous findings about one or several of these genes (Küster et al., 2004, 2007; Hohnjec et al., 2005; Valot et al., 2006; Paradi et al., 2010). However, the high number of blue copper proteins whose transcript levels changed in our experiments remains intriguing.

Recently the roadmap of cell-specific gene expression during all symbiotic stages between *M. truncatula* and *G. intraradices* (*R. irregularis*) has been deeply investigated by Hogekamp and Kuster (2013). Although the mycorrhized root tissues used in our study were most likely hosting at the same time different stages from pre-contact to senescent arbuscules in cortical cells, the transcriptional patterns of numerous DEGs, representative of various gene families, are in accordance with the work of Hogekamp and Kuster (2013). Similarly to the transcripts analysis during the stage of arbuscule formation (stage IV) we observed important transcriptional changes in genes related to posttranslational regulation, membrane transporters, protein turnover, cell wall rearrangement and defense mechanisms.

Mycorrhizal colonization of plant roots results in an extensive reorganization of cellular structures and alterations of several metabolic pathways. These changes require differential gene expression, a process primarily mediated by transcriptional regulators. In accordance with this, we identified several transcription factors, with 67, 31, and 25 up-regulated genes in +S, −S and both conditions, respectively. These data are in accordance with several works, where an up-regulation of several transcription factors was observed upon mycorrhization (Liu et al., 2003; Küster et al., 2004; Manthey et al., 2004; Sanchez et al., 2004; Hohnjec et al., 2005).

Other DEG groups were also represented by several or many members, such as the genes encoding receptor or receptor-kinase proteins (54, 29, and 23 up-regulated genes in +S, −S and both), lectins (21, 15, and 14), transporters (46, 28, and 22), proteinase inhibitors (29, 16, and 14) and lipases (21, 5, and 5).

Several genes encoding receptor-kinase proteins and lectins had already been reported (Wulf et al., 2003; Küster et al., 2004; Hohnjec et al., 2005; Manthey et al., 2004). These genes, described as related to different aspects of plant signal perception and transduction, could be the molecular effectors that mediate perception and signaling between symbionts.

Among the genes related to cell wall degradation and modification, our data confirm the findings of Hohnjec et al. (2005), with a fair presence of Myc-inducible genes, including genes encoding Pro-rich proteins (3, 3, and 2), glucanases (5, 1, and 1) and polygalacturonases (10, 2, and 2). Their regulation upon mycorrhization is consequent to the fact that these enzymes might be needed to support the extensive differentiation of membrane and cell wall structures during fungal colonization of roots and arbuscule formation.

The high amounts of proteinase inhibitors we found up-regulated upon mycorrhization are in contrast with Hohnjec et al. (2005), who only identified 6 of them, and with previous works in which even fewer genes were found associated to AM (Liu et al., 2003; Brechenmacher et al., 2004). These genes might be involved in defense mechanisms and cell regeneration processes which occur after arbuscule degradation. Our plants did not face pathogenic attacks that would have justified the induction of proteinase inhibitors related to defense mechanisms, therefore their up-regulation might indicate a high colonization rate and an intense arbuscule turnover occurring in root tissues.

Supporting the hypothesis of an intense arbuscule turnover, several proteinases and lipases were up-regulated upon mycorrhization in our +S and −S plants. As recently investigated in rice, arbuscules are temporary interchange structures between the symbionts days formed preferentially in non-colonized cortical cells and with a life span of 3–7 (Kobae and Fujiwara, 2014). Therefore, building and degradation of periarbuscular membranes and fungal residues are constant processes occurring throughout the AM interaction. Our findings confirm the need for active fatty acid and protein biosynthesis and degradation to support symbiosis. Moreover, the up-regulation of cysteine proteinases in mycorrhized roots of *M. truncatula* was also reported (Liu et al., 2003; Manthey et al., 2004), although in their work only two proteinases were induced. Both authors suggest that the AM-Specific cysteine proteinase may be involved in arbuscule senescence and in the recycling of fungal tissues, similarly to a nodule-Specific cysteine proteinase reported by Naito et al. (2000).

Mycorrhizal symbiosis plays a critical role for plant nutrient use efficiency, especially with regard to nitrogen and phosphate (Smith and Read, 2008). Efficient mycorrhizal interactions depend on the ability of the mycobiont to take nutrients available under an inorganic and/or organic form in the soil and translocate them to the host plant. In turn, organic C derived from photosynthesis is transferred from the plant to the fungus, which acts as a sink site (Bago et al., 2003). Is under these evidences that a differential regulation of transporter-related genes might be expected upon mycorrhization. Our experimental setup allowed indeed to identify 46 transporters up-regulated in the root tissues of +S plants and 28 in the root tissues of −S plants.

Among the most strongly induced genes, we identified several ABC transporters (11 in +S and 9 in −S conditions), which transport a wide variety of substrates across extra- and intracellular membranes, including metabolic products, lipids and sterols.

In contrast with Manthey et al. (2004) and Hohnjec et al. (2005), different sugar transporters (hexose, saccharose, and mannose) displayed reduced transcript accumulation. Other sugar transporters were probably recruited during specific stages of the AM interaction, but affected below the 2 fold change threshold we used to treat our data.

Different nitrate transporters were induced in both +S and −S conditions, indicating their active role in N exchanges, while four members of this gene family were down-regulated according to Hohnjec et al. (2005). Similarly, several peptide transporter gene members were up-regulated in our experiments.

Noteworthy, four zinc transporter genes were up-regulated in +S and two in −S conditions. This suggests an increased need for this metal directly dependent on the availability of other nutrients such as S. In fact, zinc is an essential component of several hundred enzymes including RNA polymerase, alkaline phosphatase, alcohol dehydrogenase, Cu/Zn superoxide dismutase, and carbonic anhydrase (Guerinot, 2000); we identified many of them as differentially affected upon mycorrhization. Besides zinc transporters of the ZIP family are implied in zinc homeostasis (Kobae et al., 2004) and also iron and manganese transport across membranes.

The Medicago sulfate transporter MtSULTR1.2 was up-regulated in the roots of −S plants, confirming our previous works on the expression of sulfate transporters using a qPCR approach (Casieri et al., 2012). Although we previously described MtSULTR1.1 and 1.2 as Myc-affected in roots and displaying different transcript accumulation levels (almost 10-fold higher for MtSULTR1.1), in this work MtSULTR1.1 did not show up among −S DEGs, and the 2 genes were absent from +S DEGs. These discrepancies might be due to a lower resolution capability of the microchip approach compared to the qPCR one. In a similar way, our results are only partially in accordance with the findings of Giovannetti et al. (2014), who described the Myc-inducible up-regulation of the sulfate transporter LjSultr1;2 (*Lotus japonicus* homolog of MtSULTR1.2), when plants were cultivated in S-Sufficient conditions.

Glutathione S-transferase (GST) was another gene family represented by several exclusively up-regulated genes in root tissues upon AM interaction. These results are in accordance with Dixon et al. (1997) who reported the root localization of ZmGSTF2 in maize (*Zea mays*). The cellular functions of some GSTs have been deeply investigated, but the function of many of them still remains poorly understood. In secondary metabolism, GSTs are involved in toxin detoxification by conjugating with glutathione (GSH), and/or reallocating flavonoid pigments. But due to their capability to act as glutathione peroxidases, antioxidants and inducers of signal molecule synthesis, GSTs are also involved in stress metabolism (Dixon et al., 2002). GSTs additionally play a key role in the deposition of flavonoid-derived pigments in maize and Petunia (Edwards et al., 2000) and are involved in the intracellular binding and stabilization of flavonoids (Mueller et al., 2000).

In conclusion, this work attempts to shed some light on the extensive transcriptional changes that occur in different biological pathways in response to S deficiency or mycorrhizal interaction. Although the picture of plant stress responses is far from being complete, our results may help focus the attention on so far neglected genes in metabolic pathways, or increase our knowledge on the extremely complex homeostasis and regulatory mechanisms used by the plant to overcome nutritional deficiencies. Several pathways appeared to be differentially affected upon S deficiency between NM and Myc plants. Particularly interesting is the fact that those pathways in which S plays an important role (i.e.,: ascorbate, glutathione and phenyl-propanoids pathways) presented a reduced amount of DEGs in mycorrhized plants. These evidences would confirm a reduced S stress compared to non-colonized plants, probably due to the increased S availability as previously reported (Casieri et al., 2012).

Despite the S deficiency stress lessening, the transcriptional responses to mycorrhizal interaction still appear to be affected by the nutritional status of the plants. This was highlighted by the lower amount of DEGs upon mycorrhization observed in plants grown in conditions of S deficiency.

The recently available genomic and transcriptomic data on *Rhizophagus irregularis* (Tisserant et al., 2013, 2012) will most probably allow to understand, from a fungal perspective, which are the mechanisms by which the plant gather S compounds during the AM interaction.

### Conflict of interest statement

The authors declare that the research was conducted in the absence of any commercial or financial relationships that could be construed as a potential conflict of interest.

## Supplementary material

Figure S1 **GO annotation (A) and GO level (B) distribution of *Medicago truncatula* genes (genome annotation Mt3.5)**. P, biological process; F, molecular function; C, cellular component.

Figure S2 **Genes differentially expressed upon S starvation in the leaves of NM and Myc Medicago plants**. Amount of DEGs and associated GO IDs are reported for each condition. Biological process **(A)**, Cellular component **(B)** and Molecular function **(C)** categories are represented as percentage of the total GO identifiers.

Figure S3 **Genes differentially expressed upon mycorrhization in the leaves of Medicago plants grown in +S and −S conditions**. Amount of DEGs and associated GO IDs are reported for each condition. Biological process **(A)**, Cellular component **(B)** and Molecular function **(C)** categories are represented as percentage of the total GO identifiers.

Figure S4 **Genes differentially expressed upon S starvation in the roots of NM and Myc Medicago plants**. Amount of DEGs and associated GO IDs are reported for each condition. Biological process **(A)**, cellular component **(B)**, and molecular function **(C)** categories are represented as percentage of the total GO identifiers.

Figure S5 **Genes differentially expressed upon mycorrhization in the roots of Medicago plants grown in +S and −S conditions**. Amount of DEGs and associated GO IDs are reported for each condition. Biological process **(A)**, cellular component **(B)**, and molecular function **(C)** categories are represented as percentage of the total GO identifiers.

Figure S6 **Distribution of differentially expressed genes upon S deficiency in the metabolic overview from the MapMan software. (A,B)** DEGs in leaves of NM and Myc plants, respectively. **(C,D)** DEGs in roots of NM and Myc plants, respectively. Twenty four out of 125 identified DEGs are represented in **(A)**, 39 out of 230 in **(B)**, 196 out of 2046 in **(C)** and 63 out of 551 in **(D)**.

Figure S7 **Distribution of differentially expressed genes upon mycorrhizal interaction in in the metabolic overview from the MapMan software**. **(A,B)** DEGs in leaves of +S and −S plants, respectively. **(C,D)** DEGs in roots of +S and −S plants, respectively. Nine out of 66 identified DEGs are represented in **(A)**, 22 out of 128 in **(B)**, 102 out of 891 in **(C)** and 14 out of 66 in **(D)**.

Table S1 **Genes differentially expressed upon mycorrhization commonly found in the roots of +S and −S grown plants**. Gene IDs, fold changes compared to NM plants, putative annotations and associated InterProScan IDs are reported.

Table S2 **Genes differentially expressed upon S starvation in the leaves of NM and Myc plants**. Gene IDs, fold changes compared to +S conditions, putative annotations, associated InterProScan IDs and KEGG enzyme codes are reported.

Table S3 **Genes differentially expressed upon mycorrhization in the leaves of +S and −S grown plants**. Gene IDs, fold changes compared to NM plants, putative annotations, associated InterProScan IDs and KEGG enzyme codes are reported.

Table S4 **Genes differentially expressed upon S starvation in the roots of NM and Myc plants**. Gene IDs, fold changes compared to +S conditions, putative annotations, associated InterProScan IDs and KEGG enzyme codes are reported.

Table S5 **Genes differentially expressed upon mycorrhization in the roots of +S and −S grown plants**. Gene IDs, fold changes compared to NM plants, putative annotations, associated InterProScan IDs and KEGG enzyme codes are reported.

## References

[B1] Bago B. Pfeffer P. E. Abubaker J. Jun J. Allen J. W. Brouillette J.. (2003). Carbon export from arbuscular mycorrhizal roots involves the translocation of carbohydrate as well as lipid. Plant Physiol. 131, 1496–1507. 10.1104/pp.102.007765 12644699 PMC166909

[B2] Bargmann B. O. Munnik T. (2006). The role of phospholipase D in plant stress responses. Curr. Opin. Plant Biol. 9, 515–522. 10.1016/j.pbi.2006.07.011 16877031

[B3] Benedito V. A. Li H. Dai X. Wandrey M. He J. Kaundal R.. (2010). Genomic inventory and transcriptional analysis of *Medicago truncatula* transporters. Plant Physiol. 152, 1716–1730. 10.1104/pp.109.148684 20023147 PMC2832251

[B4] Benedito V. A. Torres-Jerez I. Murray J. D. Andriankaja A. Allen S. Kakar K.. (2008). A gene expression atlas of the model legume *Medicago truncatula*. Plant J. 55, 504–513. 10.1111/j.1365-313X.2008.03519.x 18410479

[B5] Bouché N. Yellin A. Snedden W. A. Fromm H. (2005). Plant-specific calmodulin-binding proteins. Annu. Rev. Plant Biol. 56, 435–466. 10.1146/annurev.arplant.56.032604.144224 15862103

[B6] Brechenmacher L. Weidmann S. van Tuinen D. Chatagnier O. Gianinazzi S. Franken P.. (2004). Expression profiling of upregulated plant and fungal genes in early and late stages of *Medicago truncatula-Glomus mosseae* interactions. Mycorrhiza 14, 253–262. 10.1007/s00572-003-0263-4 13680319

[B7] Casieri L. Ait Lahmidi N. Doidy J. Fourrey C. Migeon A. Bonneau L.. (2013). Biotrophic transportome in mutualistic plant-fungal interactions. Mycorrhiza 23, 597–625. 10.1007/s00572-013-0496-9 23572325

[B8] Casieri L. Gallardo K. Wipf D. (2012). Transcriptional response of *Medicago truncatula* sulfate transporters to arbuscular mycorrhizal symbiosis with and without sulphur stress. Planta 235, 1431–1447. 10.1007/s00425-012-1645-7 22535379

[B9] Conesa A. Götz S. Garcia-Gomez J. M. Terol J. Talon M. Robles M. (2005). Blast2GO: a universal tool for annotation, visualization and analysis in functional genomics research. Bioinformatics 21, 3674–3676. 10.1093/bioinformatics/bti610 16081474

[B10] Davey M. P. Bryant D. N. Cummins I. Gates P. Ashenden T. W. Baxter R.. (2004). Effects of elevated CO2 on the vasculature and phenolic secondary metabolism of *Plantago maritima*. Phytochemistry 65, 2197–2204. 10.1016/j.phytochem.2004.06.016 15587703

[B11a] D'Hooghe P. Escamez S. Trouverie J. Avice J. C. (2013). Sulphur limitation provokes physiological and leaf proteome changes in oilseed rape that lead to perturbation of sulphur, carbon and oxidative metabolisms. BMC Plant Biol. 13:23. 10.1186/1471-2229-13-23 23391283 PMC3620940

[B11] Dixon D. P. Cole D. J. Edwards R. (1997). Characterisation of multiple glutathione transferases containing the GST I subunit with activities toward herbicide substrates in maize (*Zea mays*). Pestic Sci. 50, 72–82.

[B12] Dixon D. P. Lapthorn A. Edwards R. (2002). Plant glutathione transferases. Genome Biol. 3, 3004.1–3004.10. 10.1186/gb-2002-3-3-reviews3004 11897031 PMC139027

[B13] Doidy J. Grace E. Kuhn C. Simon-Plas F. Casieri L. Wipf D. (2012). Sugar transporters in plants and in their interactions with fungi. Trends Plant Sci. 17, 413–422. 10.1016/j.tplants.2012.03.009 22513109

[B14] Dubousset L. Abdallah M. Desfeux A.-S. Etienne P. Meuriot F. Hawkesford M.-J.. (2009). Remobilization of leaf S compounds and senescence in response to restricted sulphate supply during the vegetative stage of oilseed rape are affected by mineral N availability. J. Exp. Bot. 60, 3239–3253. 10.1093/jxb/erp172 19553370 PMC2718225

[B15] Edwards R. Dixon D. P. Walbot V. (2000). Plant glutathione S-transferases: enzymes with multiple functions in sickness and in health. Trends Plant Sci. 5, 193–198. 10.1016/S1360-1385(00)01601-0 10785664

[B16] Ferrol N. Pérez-Tienda J. (2009). Coordinated nutrient exchange in Arbuscular Mycorrhiza, in Mycorrhizas - Functional Processes and Ecological Impact, eds Azcón-Aguilar C. Barea J. M. Gianinazzi S. Gianinazzi-Pearson V. (Berlin; Heidelberg: Springer). 10.1007/978-3-540-87978-7_6

[B17] Foyer C. H. Noctor G. (2009). Redox regulation in photosynthetic organisms: signaling, acclimation, and practical implications. Antioxid. Redox Sign. 11, 861–905. 10.1089/ars.2008.2177 19239350

[B18] Giovannetti M. Tolosano M. Volpe V. Kopriva S. Bonfante P. (2014). Identification and functional characterization of a sulfate transporter induced by both sulfur starvation and mycorrhiza formation in *Lotus japonicus*. New Phytol. 204, 609–619. 10.1111/nph.12949 25132489

[B19] Götz S. García-Gómez J. M. Terol J. Williams T. D. Nagaraj S. H. Nueda M. J.. (2008). High-throughput functional annotation and data mining with the Blast2GO suite, Nucleic Acids Res. 36, 3420–3435. 10.1093/nar/gkn176 18445632 PMC2425479

[B20] Govindarajulu M. Pfeffer P. E. Jin H. Abubaker J. Douds D. D. Allen J. W.. (2005). Nitrogen transfer in the arbuscular mycorrhizal symbiosis. Nature 435, 819–823. 10.1038/nature03610 15944705

[B21] Guerinot M. L. (2000). The ZIP family of metal transporters. Biochim. Biophys. Acta 1465, 190–198. 10.1016/S0005-2736(00)00138-3 10748254

[B22] Guether M. Neuhäuser B. Balestrini R. Dynowski M. Ludewig U. Bonfante P. (2009). A mycorrhizal-specific ammonium transporter from *Lotus japonicus* acquires nitrogen released by arbuscular mycorrhizal fungi. Plant Physiol. 150, 73–83. 10.1104/pp.109.136390 19329566 PMC2675747

[B23] Güimil S. Chang H. S. Zhu T. Sesma A. Osbourn A. Roux C.. (2005). Comparative transcriptomics of rice reveals an ancient pattern of response to microbial colonization. Proc. Natl. Acad. Sci. U.S.A. 102, 8066–8070. 10.1073/pnas.0502999102 15905328 PMC1142390

[B24] Harrison M. J. Dewbre G. R. Liu J. Y. (2002). A phosphate transporter from *Medicago truncatula* involved in the acquisition of phosphate released by arbuscular mycorrhizal fungi. Plant Cell 14, 2413–2429. 10.1105/tpc.004861 12368495 PMC151226

[B25] Hewitt E. J. (1966). Sand and Water Culture Methods Used in the Study of Plant Nutrition, 2nd Edn. Farnham Royal: Commonwealth Agricultural Bureaux.

[B26] Hildebrandt U. Schmelzer E. Bothe H. (2002). Expression of nitrate transporter genes in tomato colonized by an arbuscular mycorrhizal fungus. Physiol. Plant. 115, 125–136. 10.1034/j.1399-3054.2002.1150115.x 12010476

[B27] Hirsch A. M. Bauer W. D. Bird D. M. Cullimore J. Tyler B. Yoder J. I. (2002). Molecular signals and receptors- controlling rhizosphere interactions between plants and other organisms-by chance or intent? Ecology 84, 858–868 10.1890/0012-9658(2003)084[0858:MSARCR]2.0.CO;2

[B28] Hogekamp C. Arndt D. Pereira P. A. Becker J. D. Hohnjec N. Kuster H. (2011). Laser microdissection unravels cell-type-specific transcription in arbuscular mycorrhizal roots, including CAAT-box transcription factor gene expression correlating with fungal contact and spread. Plant Physiol. 157, 2023–2043. 10.1104/pp.111.186635 22034628 PMC3327204

[B29] Hogekamp C. Kuster H. (2013). A roadmap of cell-type specific gene expression during sequential stages of the arbuscular mycorrhiza symbiosis. BMC Genomics 14:306. 10.1186/1471-2164-14-306 23647797 PMC3667144

[B30] Hohnjec N. Vieweg M. E. Pühler A. Becker A. Kuster H. (2005). Overlaps in the transcriptional profiles of *Medicago truncatula* roots inoculated with two different *Glomus* fungi provide insights into the genetic program activated during arbuscular mycorrhiza. Plant Physiol. 137, 1283–1301. 10.1104/pp.104.056572 15778460 PMC1088321

[B31] Iqbal N. Masood A. Iqbal M. Khan R. Asgher M. Fatma M.. (2013). Cross-talk between sulfur assimilation and ethylene signaling in plants. Plant Signal. Behav. 8, e22478 104–112. 10.4161/psb.22478 23104111 PMC3745555

[B32] Javot H. Penmetsa V. R. Breuillin F. Bhattarai K. K. Noar R. D. Gomez S. K.. (2011). *Medicago truncatula* MtPt4 mutants reveal a role for nitrogen in the regulation of arbuscule degeneration in arbuscular mycorrhizal symbiosis. Plant J. 68, 954–965. 10.1111/j.1365-313X.2011.04746.x 21848683

[B33] Javot H. Pumplin N. Harrison M. J. (2007). Phosphate in the arbuscular mycorrhizal symbiosis: transport properties and regulatory roles. Plant Cell Environ. 30, 310–322. 10.1111/j.1365-3040.2006.01617.x 17263776

[B34] Karandashov V. Bucher M. (2005). Symbiotic phosphate transport in arbuscular mycorrhizas. Trends Plant Sci. 10, 22–29. 10.1016/j.tplants.2004.12.003 15642520

[B35] Kaschuk G. Kuyper T. W. Leffelaar P. A. Hungria M. Giller K. E. (2009). Are the rates of photosynthesis stimulated by the carbon sink strength of rhizobial and arbuscular mycorrhizal symbioses? Soil Biol. Biochem. 41, 1233–1244 10.1016/j.soilbio.2009.03.005

[B36] Kobae Y. Fujiwara T. (2014). Earliest colonization events of *Rhizophagus irregularis* in rice roots occur preferentially in previously uncolonized cells. Plant Cell Physiol. 55, 1497–1510. 10.1093/pcp/pcu081 24899551

[B37] Kobae Y. Tamura Y. Takai S. Banba M. Hata S. (2010). Localized expression of arbuscular mycorrhiza-inducible ammonium transporters in soybean. Plant Cell Physiol. 51, 1411–1415. 10.1093/pcp/pcq099 20627949

[B38] Kobae Y. Uemura T. Sato M. H. Ohnishi M. Mimura T. Nakagawa T.. (2004). Zinc transporter of *Arabidopsis thaliana* AtMTP1 is localized to vacuolar membranes and implicated in zinc homeostasis. Plant Cell Physiol. 45, 1749–1758. 10.1093/pcp/pci015 15653794

[B39] Kopriva S. Mugford S. G. Matthewman C. A. Koprivova A. (2009). Plant sulfate assimilation genes: redundancy versus specialization. Plant Cell Rep. 28, 1769–1780. 10.1007/s00299-009-0793-0 19876632

[B40] Koprivova A. Suter M. den Camp R. O. Brunold C. Kopriva S. (2000). Regulation of sulfate assimilation by nitrogen in *Arabidopsis*. Plant Physiol. 122, 737–746. 10.1104/pp.122.3.737 10712537 PMC58909

[B41] Küster H. Hohnjec N. Krajinski F. El Yahyaoui F. Manthey K. Gouzy J.. (2004). Construction and validation of cDNA-based Mt6k-RIT macro- and microarrays to explore root endosymbioses in the model legume *Medicago truncatula*. J. Biotechnol. 108, 95–113. 10.1016/j.jbiotec.2003.11.011 15129719

[B42] Küster H. Vieweg M. F. Manthey K. Baier M. C. Hohnjec N. Perlick A. M. (2007). Identification and expression regulation of symbiotically activated legume genes. Phytochemistry 68, 8–18. 10.1016/j.phytochem.2006.09.029 17081575

[B43] Limpens E. Moling S. Hooiveld G. Pereira P. A. Bisseling T. Becker J. D.. (2013). Cell- and tissue-specific transcriptome analyses of *Medicago truncatula* root nodules. PLOS ONE 8:e64377. 10.1371/journal.pone.0064377 23734198 PMC3667139

[B44] Liu J. Blaylock L. A. Endre G. Cho J. Town C. D. VandenBosch K.. (2003). Transcript profiling coupled with spatial expression analyses reveals genes involved in distinct developmental stages of an arbuscular mycorrhizal symbiosis. Plant Cell 15, 2106–2123. 10.1105/tpc.014183 12953114 PMC181334

[B45] Lo S. F. Yang S. Y. Chen K.-T. Hsing Y.-I. Zeevaart J. A. D. Chen L. J.. (2008). A novel class of gibberellin 2-oxidases control semidwarfism, tillering, and root development in rice. Plant Cell 20, 2603–2618. 10.1105/tpc.108.060913 18952778 PMC2590730

[B46] Maeda D. Ashida K. Iguchi K. Chechetka S. A. Hijikata A. Okusako Y.. (2006). Knockdown of an arbuscular mycorrhiza-inducible phosphate transporter gene of *Lotus japonicus* suppresses mutualistic symbiosis. Plant Cell Physiol. 47, 807–817. 10.1093/pcp/pcj069 16774930

[B47] Manthey M. Krajinski F. Hohnjec N. Firnhaber C. Pühler A. Perlick A. M.. (2004). Transcriptome profiling in root nodules and arbuscular mycorrhiza identifies a collection of novel genes induced during *Medicago truncatula* root endosymbioses. Mol. Plant Microbe Interact. 17, 1063–1077. 10.1094/MPMI.2004.17.10.1063 15497399

[B48] Maruyama-Nakashita A. Inoue E. Watanabe-Takahashi A. Yamaya T. Takahashi H. (2003). Transcriptome profiling of sulfur-responsive genes in *Arabidopsis* reveals global effects of sulfur nutrition on multiple metabolic pathways. Plant Physiol. 132, 597–605. 10.1104/pp.102.019802 12805590 PMC167000

[B49] McGrath S. P. Zhao F. J. Withers P. J. A. (1996). Development of sulphur deficiency in crops and its treatment, in Proceedings of the Fertilizer Society 379 (St. Petersburg).

[B50] Mueller L. A. Goodman C. D. Silady R. A. Walbot V. (2000). AN9, a petunia glutathione S-transferase required for anthocyanin sequestration, is a flavonoid-binding protein. Plant Physiol. 123, 1561–1570. 10.1104/pp.123.4.1561 10938372 PMC59113

[B51] Munnik T. (2001). Phosphatidic acid: an emerging plant lipid second messenger. Trends Plant Sci. 6, 227–233. 10.1016/S1360-1385(01)01918-5 11335176

[B52] Naito Y. Fujie M. Usami S. Murooka Y. Yamada T. (2000). The involvement of a cysteine proteinase in the nodule development in Chinese milk vetch infected with *Mesohizobium huakuii* subsp. rengei. Plant Physiol. 124, 1087–1095. 10.1104/pp.124.3.1087 11080286 PMC59208

[B53] Nikiforova V. J. Freitag J. Kempa S. Adamik M. Hesse H. Hoefgen R. (2003). Transcriptome analysis of sulfur depletion in *Arabidopsis thaliana*: interlacing of biosynthetic pathways provides response specificity. Plant J. 33, 633–650. 10.1046/j.1365-313X.2003.01657.x 12609038

[B54] Ohkama N. Takei K. Sakakibara H. Hayashi H. Yoneyama T. Fujiwara T. (2002). Regulation of sulfur-responsive gene expression by exogenously applied cytokinins in *Arabidopsis thaliana*. Plant Cell Physiol. 43, 1493–1501. 10.1093/pcp/pcf183 12514246

[B55] Ohkama-Ohtsu N. Wasaki J. (2010). Recent progress in plant nutrition research: cross-talk between nutrients, plant physiology and soil microorganisms. Plant Cell Physiol. 51, 1255–1264. 10.1093/pcp/pcq095 20624893

[B56] Paradi I. van Tuinen D. Morandi D. Ochatt S. Robert F. Jacas L.. (2010). Transcription of two blue copper-binding protein isogenes is highly correlated with arbuscular mycorrhizal development in *Medicago truncatula*. Mol. Plant Microbe Interact. 23, 1175–1183. 10.1094/MPMI-23-9-1175 20687807

[B57] Parniske M. (2008). Arbuscular mycorrhiza: the mother of plant root endosymbioses. Nat. Rev. Microbiol. 6, 763–775. 10.1038/nrmicro1987 18794914

[B58] Paszkowski U. Kroken S. Roux C. Briggs S. P. (2002). Rice phosphate transporters include an evolutionarily divergent gene specifically activated in arbuscular mycorrhizal symbiosis. Proc. Natl. Acad. Sci. U.S.A. 99, 13324–13329. 10.1073/pnas.202474599 12271140 PMC130632

[B59] Popper Z. Michel G. Herve C. Domozych D. Willats W. G. T. Tuohy M. G.. (2011). Evolution and diversity of plant cell walls: from algae to flowering plants. Annu. Rev. Plant Biol. 62, 567–590. 10.1146/annurev-arplant-042110-103809 21351878

[B60] Rehman A. (2010). Does Arbuscular Mycorrhiza Symbiosis Increase the Capacity or the Efficiency of Photosynthetic Apparatus in the Model Legume Medicago truncatula? Ph.D. thesis. Linköpings universitet.

[B61] Saito K. (2004). Sulfur assimilatory metabolism. The long and smelling road. Plant Physiol. 136, 2443–2450. 10.1104/pp.104.046755 15375200 PMC523311

[B62] Sanchez L. Weidmann S. Brechenmacher L. Batoux M. van Tuinen D. Lemanceau P. (2004). Common gene expression in *Medicago truncatula* roots in response to *Pseudomonas fluorescens* colonization, mycorrhiza development and nodulation. New Phytol. 161, 855–863 10.1046/j.1469-8137.2004.00997.x 33873727

[B63] Sawers R. J. H. Yang S.-Y. Gutjahr C. Paszkowsky U. (2008). The molecular components of nutrient exchange in arbuscular mycorrhizal interactions, in Mycorrhizae: Sustainable Agriculture and Forestry, eds Siddiqui Z. A. Akhtar M. S. Futai K. (Dordrecht: Springer Science – Business Media B.V.), 37–59.

[B64] Siqueira J. O. Safir G. R. Nair M. G. (1991). Stimulation of vesicular-arbuscular mycorrhiza formation and growth of white clover by flavonoid compounds. New Phytol. 118, 87–93 10.1111/j.1469-8137.1991.tb00568.x

[B65] Smith S. E. Jakobsen I. Grønlund M. Smith F. A. (2011). Roles of arbuscular mycorrhizas in plant phosphorus nutrition, interactions between pathways of phosphorus uptake in arbuscular mycorrhizal roots have important implications for understanding and manipulating plant phosphorus acquisition. Plant Physiol. 156, 1050–1057. 10.1104/pp.111.174581 21467213 PMC3135927

[B66] Smith S. E. Read D. J. (2008). Mycorrhizal Symbiosis, 3rd Edn. London: Academic Press.

[B67] Smith S. E. Smith F. A. (2011). Roles of arbuscular mycorrhizas in plant nutrition and growth: new paradigms from cellular to ecosystem scales. Annu. Rev. Plant Biol. 62, 16.1–16.24. 10.1146/annurev-arplant-042110-103846 21391813

[B68] Smith S. E. Smith F. A. (2012). Fresh perspectives on the roles of arbuscular mycorrhizal fungi in plant nutrition and growth. Mycologia 104, 1–13. 10.3852/11-229 21933929

[B69] Sunarpi Anderson J. W. (1997a). Allocation of S in generative growth of soybean. Plant Physiol. 114, 687–693. 12223736 10.1104/pp.114.2.687 PMC158353

[B70] Sunarpi Anderson J. W. (1997b). Effect of nitrogen nutrition on remobilization of protein sulphur in the leaves of vegetative soybean and associated changes in soluble sulphur metabolites. Plant Physiol. 115, 1671–1680. 12223887 10.1104/pp.115.4.1671 PMC158633

[B71a] Thimm O. Blaesing O. Gibon Y. Nagel A. Meyer S. Krüger P.. (2004). MAPMAN: a user-driven tool to display genomics data sets onto diagrams of metabolic pathways and other biological processes. Plant J. 37, 914–939. 10.1111/j.1365-313X.2004.02016.x 14996223

[B71] Tisserant E. Kohler A. Dozolme-Seddas P. Balestrini R. Benabdellah K. Colard A.. (2012). The transcriptome of the arbuscular mycorrhizal fungus *Glomus intraradices* (DAOM 197198) reveals functional tradeoffs in an obligate symbiont. New Phytol. 193, 755–769. 10.1111/j.1469-8137.2011.03948.x 22092242

[B72] Tisserant E. Malbreil M. Kuo A. Kohler A. Symeonidi A. Balestrini R.. (2013). Genome of an arbuscular mycorrhizal fungus provides insight into the oldest plant symbiosis. Proc. Natl. Acad. Sci. U.S.A. 110, 20117–20122. 10.1073/pnas.1313452110 24277808 PMC3864322

[B73] Trouvelot A. Kough J. L. Gianinazzi-Pearson V. (1986). Mesure du taux de mycorhization VA d'un systeme radiculaire. Recherche de methodes d'estimation ayant une signification fonctionnelle, in Physiological and Genetical Aspects of Mycorrhizae. Proceedings of the 1st European Symposium on Mycorrhizae, eds Gianinazzi-Pearson V. Gianinazzi S. (Paris: Institut National de la Recherche Agronomique), 217–221.

[B74] Valot B. Negroni L. Zivy M. Gianinazzi S. Dumas-Gaudot E. (2006). A mass spectrometric approach to identify arbuscular mycorrhiza-related proteins in root plasma membrane fractions. Proteomics 6, S145–S155. 10.1002/pmic.200500403 16511816

[B75] Vierheilig H. Coughlan A. P. Wyss U. Piché Y. (1998). Ink and vinegar, a simple staining technique for arbuscular-mycorrhizal fungi. Appl. Environ. Microb. 64, 5004–5007. 9835596 10.1128/aem.64.12.5004-5007.1998 PMC90956

[B76] Wulf A. Manthey K. Doll J. Perlick A. M. Linke B. Bekel T.. (2003). Transcriptional changes in response to arbuscular mycorrhiza development in the model plant *Medicago truncatula*. Mol. Plant Microbe Interact. 16, 306–314. 10.1094/MPMI.2003.16.4.306 12744459

[B77] Zhang W. Wan X. Hong Y. Li W. Wang X. (2009). Plant phospholipase D. Lipid signaling in plants. Plant Cell Monogr. 16, 39–62 10.1007/978-3-642-03873-0_3

