# Supplementary figures and images for: Transcriptional responses of Medicago truncatula upon sulfur deficiency stress and arbuscular mycorrhizal symbiosis
Source: Front Plant Sci. 2014 Dec 2;5:680. doi: 10.3389/fpls.2014.00680 (PMC4251294; doi:10.3389/fpls.2014.00680)

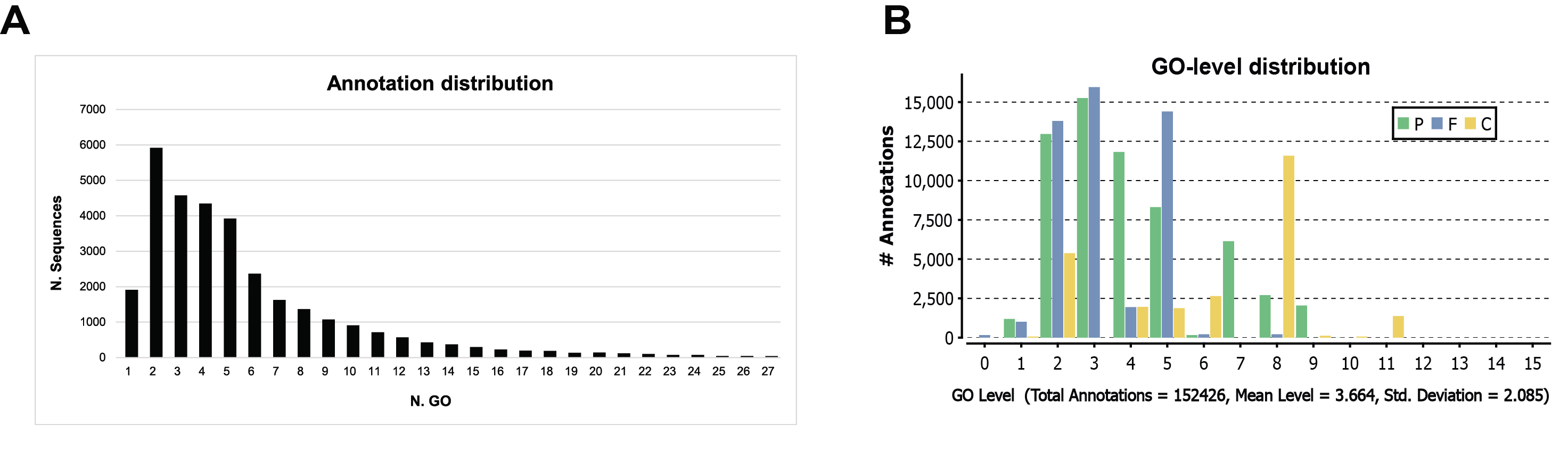

Supplement: Figure S1 — GO annotation (A) and GO level (B) distribution of Medicago truncatula genes (genome annotation Mt3.5). P, biological process; F, molecular function; C, cellular component. [file DataSheet1.ZIP › Supplementary Material/Figure S1.JPEG]

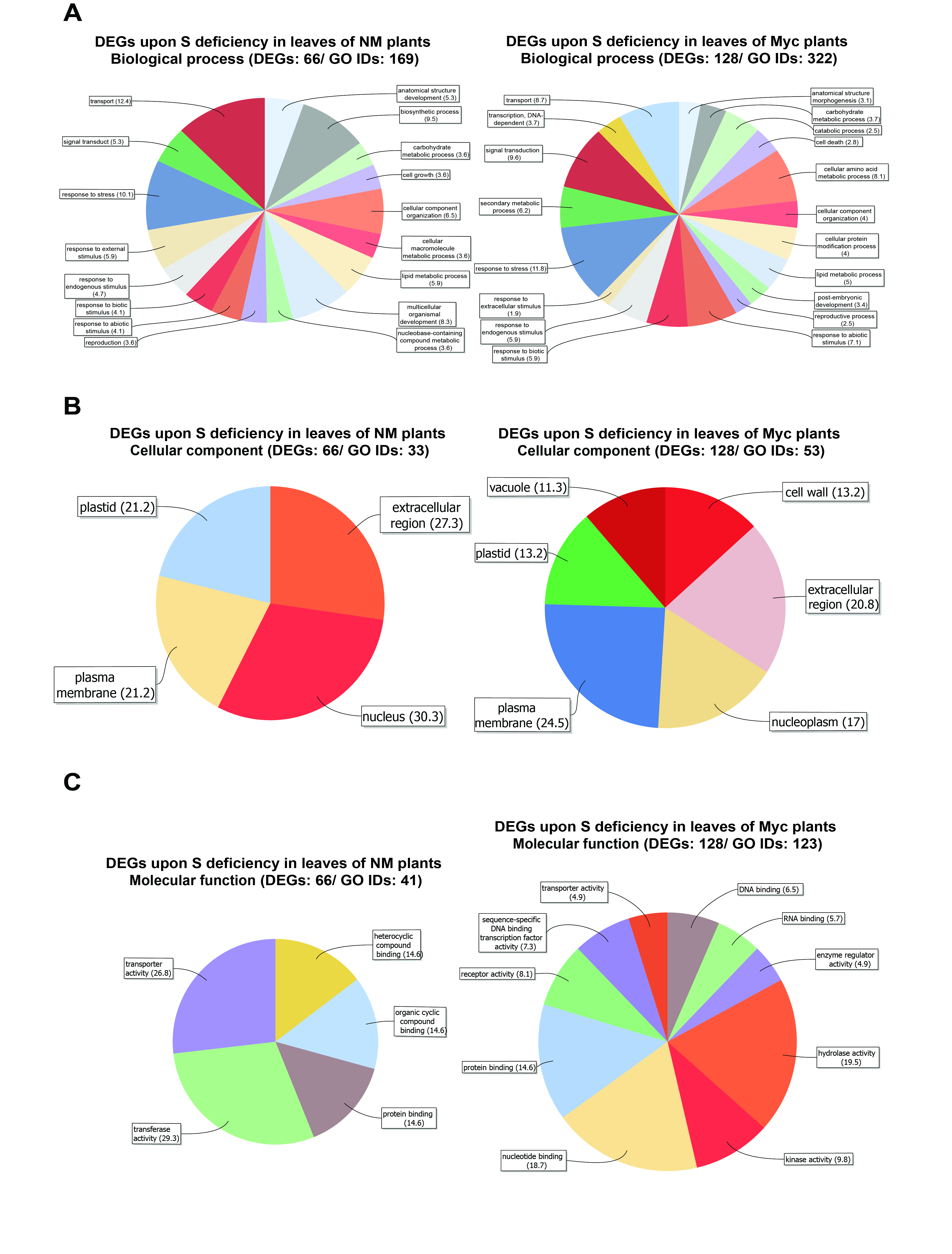

Supplement: Figure S1 — GO annotation (A) and GO level (B) distribution of Medicago truncatula genes (genome annotation Mt3.5). P, biological process; F, molecular function; C, cellular component. [file DataSheet1.ZIP › Supplementary Material/Figure S2.JPEG]

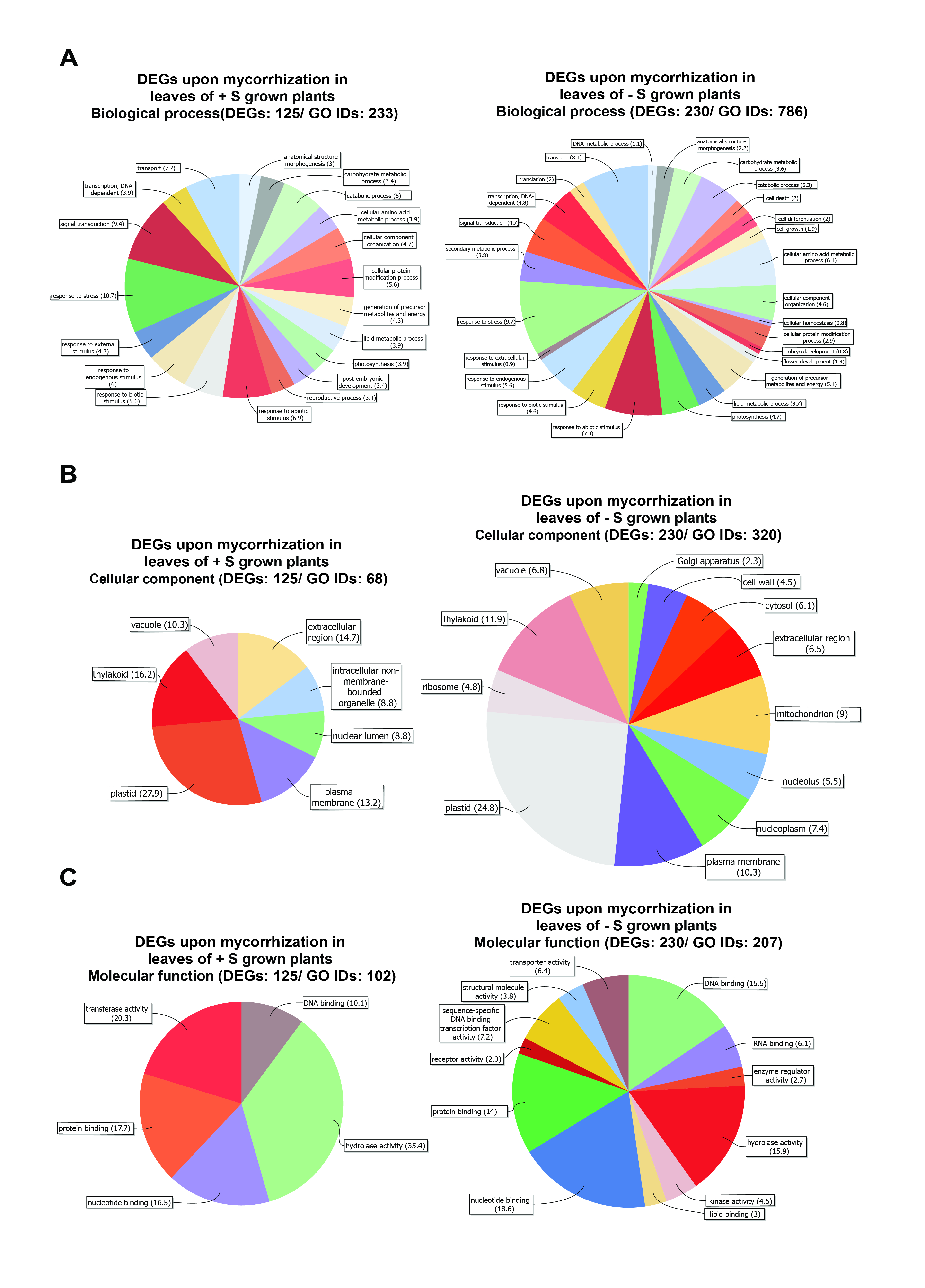

Supplement: Figure S1 — GO annotation (A) and GO level (B) distribution of Medicago truncatula genes (genome annotation Mt3.5). P, biological process; F, molecular function; C, cellular component. [file DataSheet1.ZIP › Supplementary Material/Figure S3.JPEG]

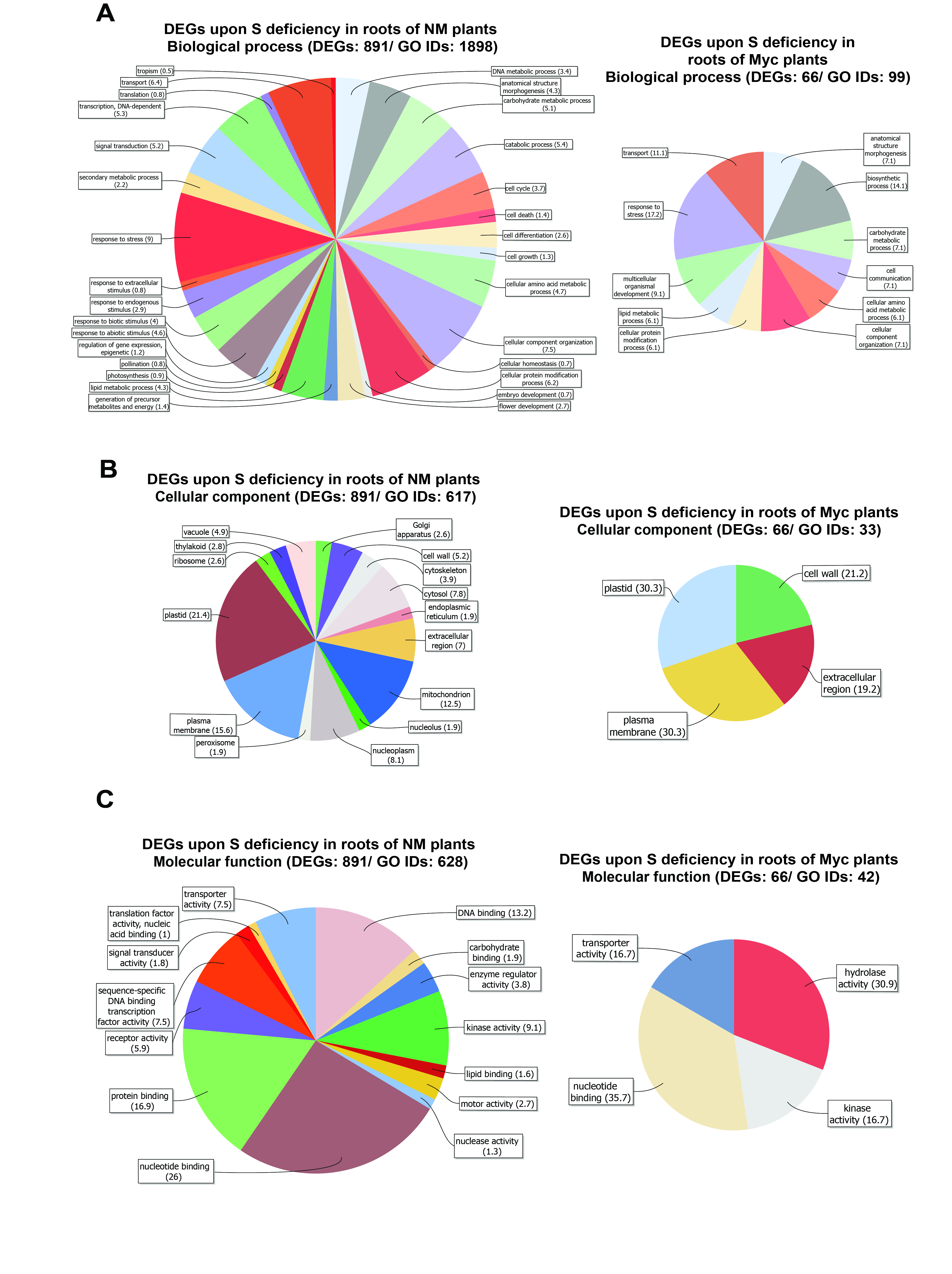

Supplement: Figure S1 — GO annotation (A) and GO level (B) distribution of Medicago truncatula genes (genome annotation Mt3.5). P, biological process; F, molecular function; C, cellular component. [file DataSheet1.ZIP › Supplementary Material/Figure S4.JPEG]

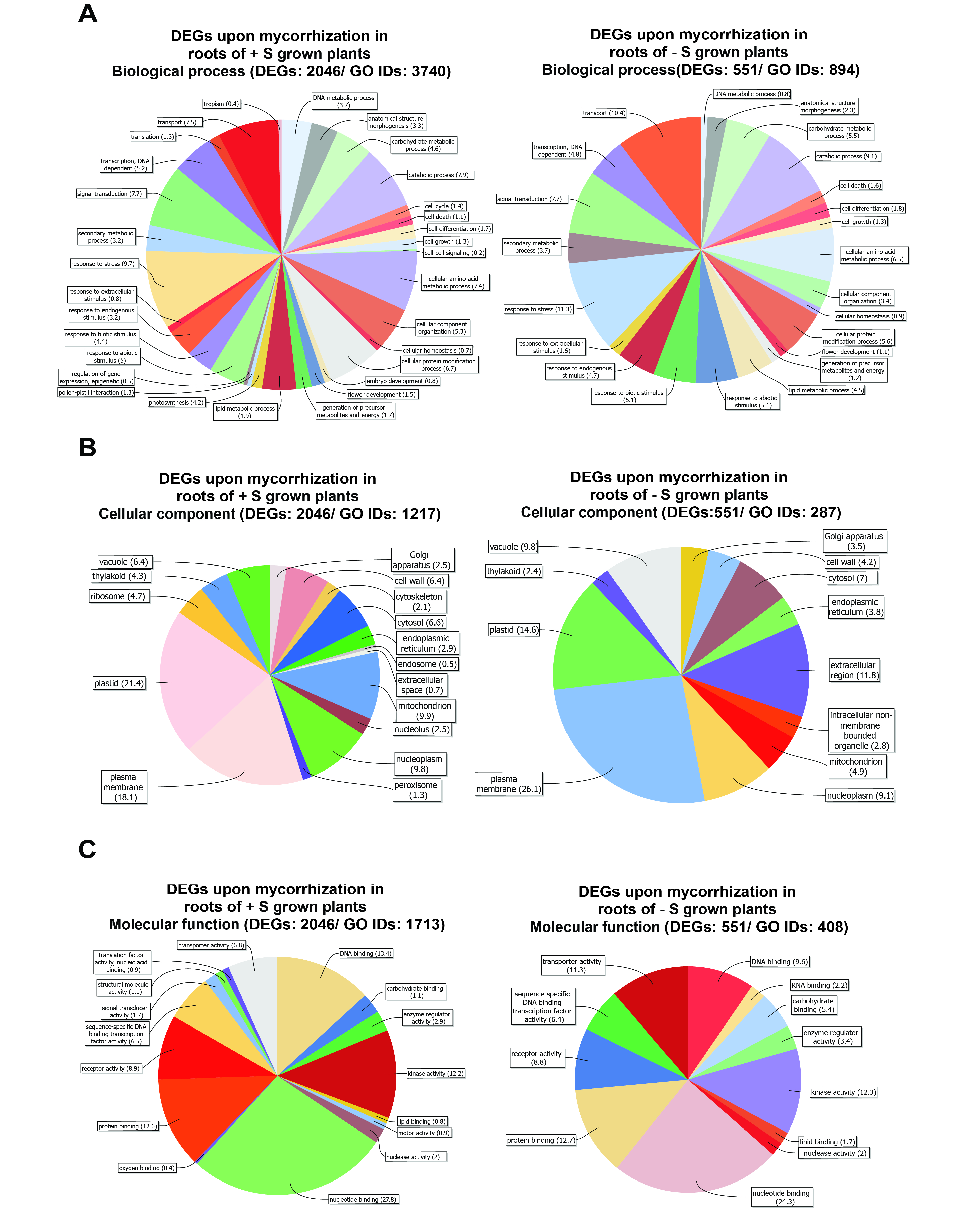

Supplement: Figure S1 — GO annotation (A) and GO level (B) distribution of Medicago truncatula genes (genome annotation Mt3.5). P, biological process; F, molecular function; C, cellular component. [file DataSheet1.ZIP › Supplementary Material/Figure S5.JPEG]

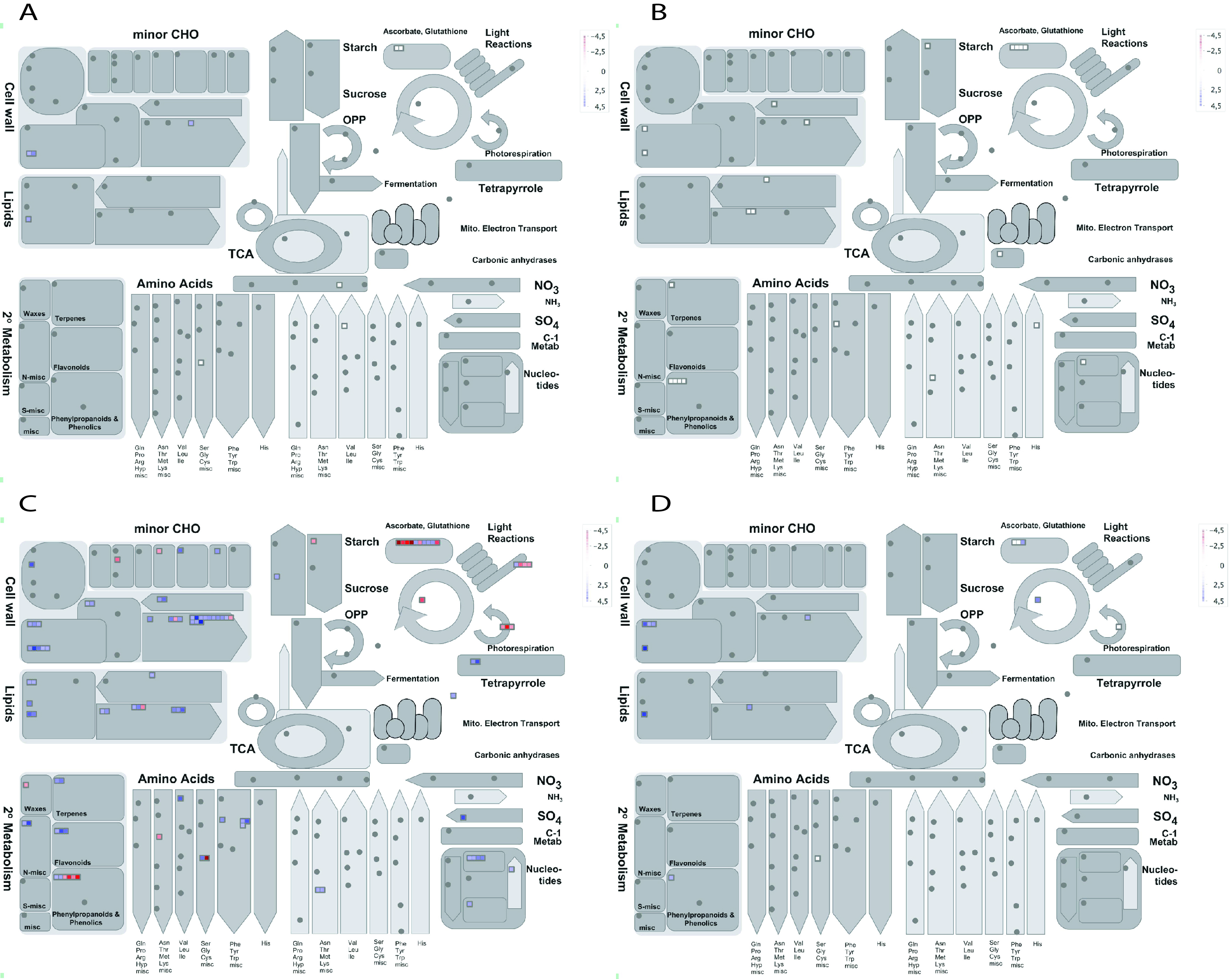

Supplement: Figure S1 — GO annotation (A) and GO level (B) distribution of Medicago truncatula genes (genome annotation Mt3.5). P, biological process; F, molecular function; C, cellular component. [file DataSheet1.ZIP › Supplementary Material/Figure S6.JPEG]

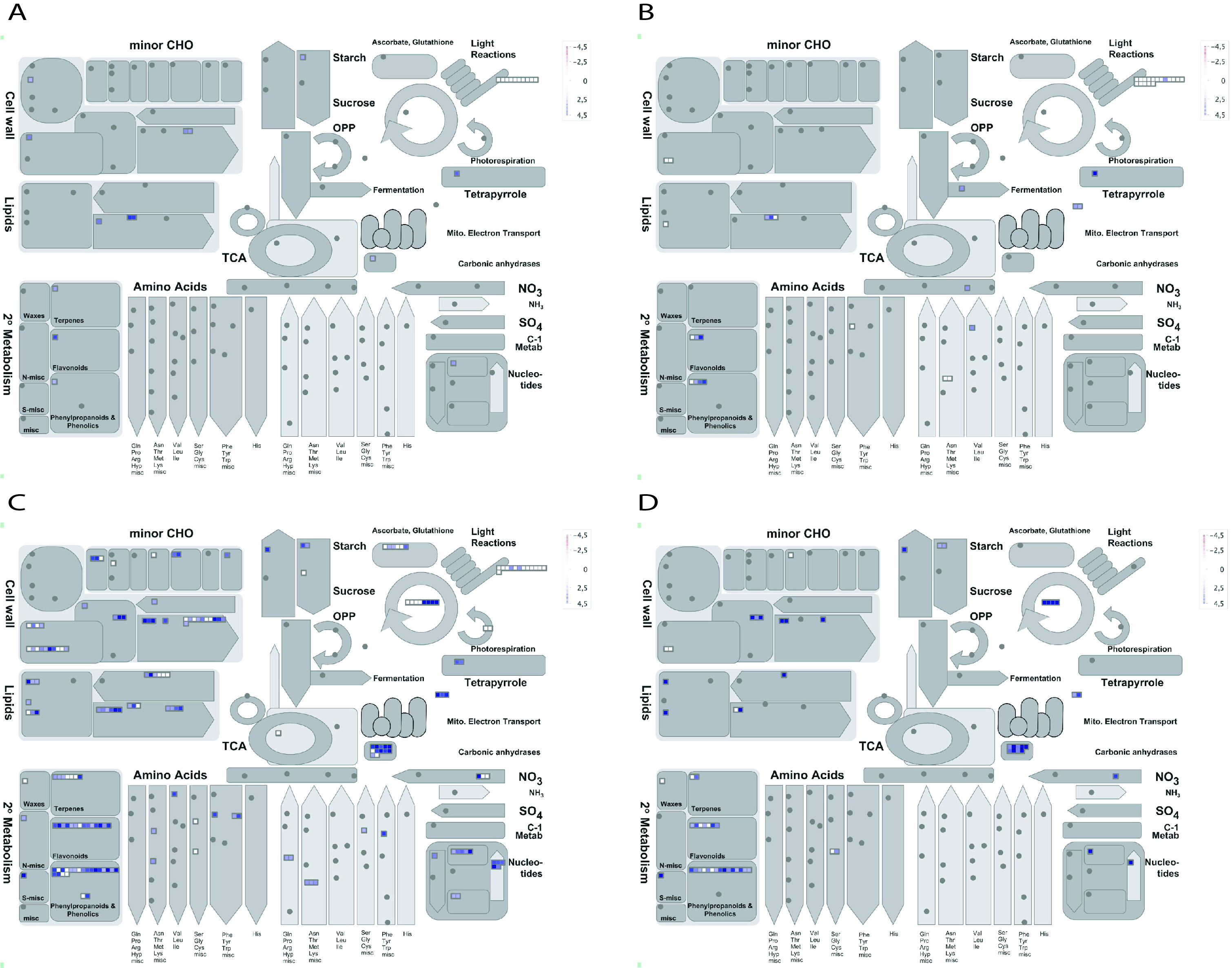

Supplement: Figure S1 — GO annotation (A) and GO level (B) distribution of Medicago truncatula genes (genome annotation Mt3.5). P, biological process; F, molecular function; C, cellular component. [file DataSheet1.ZIP › Supplementary Material/Figure S7.JPEG]
